# Genotype Analysis on *Orientia tsutsugamushi* Causing Scrub Typhus in Malaysia: A Re-Emerging Disease

**DOI:** 10.3390/tropicalmed10090252

**Published:** 2025-09-02

**Authors:** Shirley Yi Fen Hii, Maswani Nabilah Mohd Zaidi, Wan Norazanin Kassim, Rohaidah Hashim, Siti Roszilawati Ramli

**Affiliations:** Bacteriology Unit, Infectious Diseases Research Centre, Institute for Medical Research, National Institutes of Health, Ministry of Health Malaysia, Persiaran Setia Murni, Setia Alam, 40170 Shah Alam, Selangor, Malaysia; shirley.hy@moh.gov.my (S.Y.F.H.); maswaninabilah9@moh.gov.my (M.N.M.Z.); norazanin.kassim@moh.gov.my (W.N.K.); rohaidah@moh.gov.my (R.H.)

**Keywords:** acute scrub typhus, *Orientia tsutsugamushi*, Malaysia, genotypes, PCR, *tsa56*

## Abstract

**Introduction:** Scrub typhus is caused by Gram-negative bacteria, *Orientia tsutsugamushi*. Humans are the dead-end host of scrub typhus. Currently, there is no vaccine available. The disease can be fatal without appropriate treatment. Here, we present the circulating OT genotypes in Malaysia and a *tsa56*-based single PCR to detect and determine OT genotypes, which is an approach to replace the time-consuming traditional nested PCR. **Methods:** The patients’ blood or tissue samples (*n* = 1200), received from all hospitals in Malaysia from December 2022 to November 2024, were screened for rickettsial infections. Both *htr*A qPCR and nested PCR were performed to detect the presence of OT DNA. Simultaneously, a selection of DNA was evaluated for the new single PCR protocol and confirmed with Sanger sequencing. **Results:** We report that Pahang state of Peninsular Malaysia presents the highest number of acute scrub typhus infections in Malaysia within the 24 months period. There are four genotypes circulating in the Malaysian population. OT genotype Gilliam (*n* = 31, 29.2%) and Karp (*n* = 31, 29.2%) are the predominant OT genotypes in Malaysia, followed by TA763 (*n* = 22, 20.8%) and Kato (*n* = 22, 20.8%). The single-run PCR presents longer sequence size and similar results with the nested PCR. **Conclusions:** Acute scrub typhus infection is not rare in Malaysia and should be considered for undifferentiated febrile illness. The single-run PCR protocol is time-saving and a promising approach for OT detection and genotype analysis in a single run to complement a clinical diagnostic setting and surveillance.

## 1. Introduction

Scrub typhus is caused by obligate intracellular Gram-negative bacteria, *Orientia tsutsugamushi* (OT). The disease is widely distributed across the Asia-Pacific region covering Russia (north), Pakistan (west), Australia (south), and Japan (east) [1]. Globally, it is estimated that more than a million cases occur annually and a billion people are at risk [2]. It is one of the major causes of acute febrile illness (AFI) in tropical regions like Southeast Asia [3,4]. Currently, there is no commercial vaccine available against scrub typhus. Doxycycline remains the effective antibiotic treatment for scrub typhus. The case fatality rate can reach up to 70% without appropriate treatment [1]. Malaysia has had a long history of scrub typhus since 1926, as reported by Fletcher and Field [5]. Despite recognized as a scrub typhus endemic area, there is limited information on the incidence of scrub typhus in Malaysia. Data from seroprevalence studies and case reports spanning the last three decades support the persistent endemicity of scrub typhus in the local community [4,6,7,8]. Recent studies in Sabah, Perak, and Negeri Sembilan showed that among the rickettsial illnesses, scrub typhus has been reported as the most frequent infection among febrile hospitalized patients in Malaysia [4,7,8]. In addition, increasing incidence have been observed in neighbouring countries including China, Sri Lanka, and Thailand [9].

The 56 kDa type-specific antigen (TSA56) is the most abundant surface protein of OT. The variability in *tsa56* gene (VDI, VDII, VDIII, VDIV) accounts for the difference in OT, resulting in the assignment of different genotypes in OT [10,11]. By the end of 2015, more than a thousand TSA sequences were deposited in the public database. High mutation rate and recombination are observed, resulting in expansion of the genotypes from the prototype Karp, Kato, and Gilliam genotypes. Currently, there are more than 30 serotypes for OT. Regional distribution of OT genotypes was observed [9]. *tsa56* gene sequence is commonly used for phylogenetic analysis to observe the distribution of *O. tsutsugamushi* genotypes in different geographical regions [9,10,11,12,13]. In this study, we aim to determine the circulating and the predominant OT genotypes in Malaysia. In addition, we also evaluate a single-run PCR procedure compared to the commonly used nested PCR.

## 2. Materials and Methods

### 2.1. DNA Extraction and PCR

The suspected rickettsial infection samples from December 2022 to November 2024 (*n* = 1200) were received from all hospitals in Malaysia to screen for acute rickettsial diseases. The cases include fever within 10 days duration after onset with or without presence of eschar and other clinical features. They may have positive history of contact with rodents or animals; exposure history to insect bites; or working at a rubber estate or oil palm plantation. The samples were of patients’ blood, eschars, urine, or cerebrospinal fluid (CSF) received by the Institute of Medical Research for diagnostics of acute scrub typhus infection. First, the DNA was extracted from buffy coat (EDTA blood) or tissue using a QIAmp^®^ DNA Blood Mini Kit (Qiagen, Hilden, Germany). Both real-time quantitative PCR (qPCR) and nested PCR (nPCR) were performed. In qPCR, the 47 kDa periplasmic serine protease *htr*A (47 kDa) is targeted and prepared as described [14,15]. Briefly, a total of 25 µL reaction per tube was prepared, including 6.25 µL master mix and 1 µL of each primer (forward: 5′-AACTGATTTTATTCAAAC TAATGCTGC T-3′, reverse: 5′-TATGCCTGAGTAAGATACRTGAATRGAATT-3′) and probe (6FAM-TGGGTAGCTTTGGTGGACCGATGTTTAATCT-TAMRA). The two-step qPCR reaction was performed on a QuantStudio^TM^ 6 Flex Real-Time PCR system (Thermo Fisher Scientific, Waltham, MA, USA) at initial denaturation; 94 °C for 5 min followed by 40 cycles of denaturation at 94 °C, 5 s, and annealing at 60 °C, 30 s using a 4X CAPITAL^TM^ qPCR Probe Master Mix (Biotech Rabbit, Berlin, Germany) according to the manufacturer’s protocol.

The qPCR-positive DNA were proceeded for nested PCR to determine the genotypes of the OT strains. Two rounds of PCR were performed in nested PCR using primers: (i) outer (forward: 5′-TCAAGCTTATTGCTAGTGCAATGTCTGC-3′, reverse: 5′-AGGGATCCCTGCTGCTGTGCTTGCTGCG-3′); (ii) inner (forward:5′-GATCAAGCTTCCTCAGCCTACTATAATGCC-3′, reverse: 5′-CTAGGGATCCCGACAGATGCACTATTAGGC-3′) [16]. First, the outer fragments of *tsa56* were amplified followed by the amplification of the inner fragments using a DNA template and the first PCR product, respectively. The PCR was run using PCRBIO Ultra mix (PCR Biosystems, Wayne, PA, USA) and the same conditions for both rounds of PCR: initial denaturation at 94 °C for 2 s, denaturation at 94 °C for 30 s, annealing at 57 °C for one min and extension at 72 °C for one min for 35 cycles and final extension at 72 °C for 10 min. The PCR product was run on a 2% agarose gel and viewed on Bio-Rad ChemiDoc Touch Image System (Bio-Rad, Hercules, CA, USA).

### 2.2. Single-Run PCR

The single-run PCR protocol used one set of primers, TSA1F (5′-AGTTTAGAATGGTTA CCACTA-3′) and TSA1R (5′-CTGCATGACGCTGCAATTT-3′). The reaction mixture contained 12.5 µL of Q5^®^ High-Fidelity 2X Master Mix (New England Biolabs, Ipswich, MA, USA) and 10 pmol of each primer, with the PCR conditions as follows: 98 °C for 30 s (polymerase activation), 40 cycles of denaturation, annealing and extension at 98 °C for 10 s, 56 °C for one min, and 72 °C for 30 s with a final extension at 72 °C for 2 min. OT DNA ranging from 0.02 ng/µL to 10 ng/µL were tested using a single-run PCR protocol. The PCR products were run on a 1.5% agarose gel at 80 V, 45 min and viewed on Bio-Rad ChemiDoc Touch Image System (Bio-Rad, Hercules, CA, USA).

### 2.3. OT Genotyping

The PCR products were sequenced by Sanger sequencing (1st Base, Seri Kembangan, Malaysia). The sequences were first blasted against NCBI databases to check completeness and the correct CDS region. The blast results were used as a guide for the selection of reference genes for phylogenetic analysis. All the *tsa56* partial genes from nested PCR and TSA1 sequences were aligned together with other known OT genotype strains (Table S1), and a phylogenetic tree was generated using the maximum likelihood method conducted in MEGA 11 [17]. Bootstrap analysis with 1000 repetitions was performed to assess the robustness and reliability of the tree branching.

### 2.4. Ethics Statement

This study was registered with the National Medical Research Register (NMRR) and ethically approved by the Medical Research and Ethics Committee (MREC), Ministry of Health, Malaysia with reference numbers NMRR ID-23-01678-ITL and NMRR ID-24-02585-ZD9. All samples included in this study were post-diagnostic specimens collected as part of routine clinical care and processed according to standard protocols. Prior to inclusion in the study, all samples were de-identified and anonymized to ensure patient confidentiality. As such, the requirement for informed consent was waived.

## 3. Results

**Figure 1:**
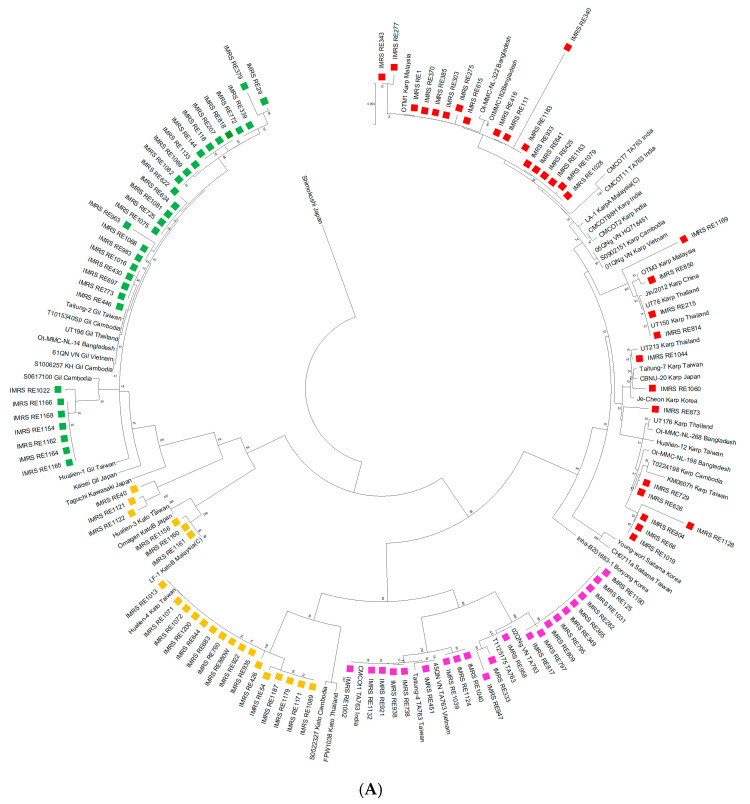

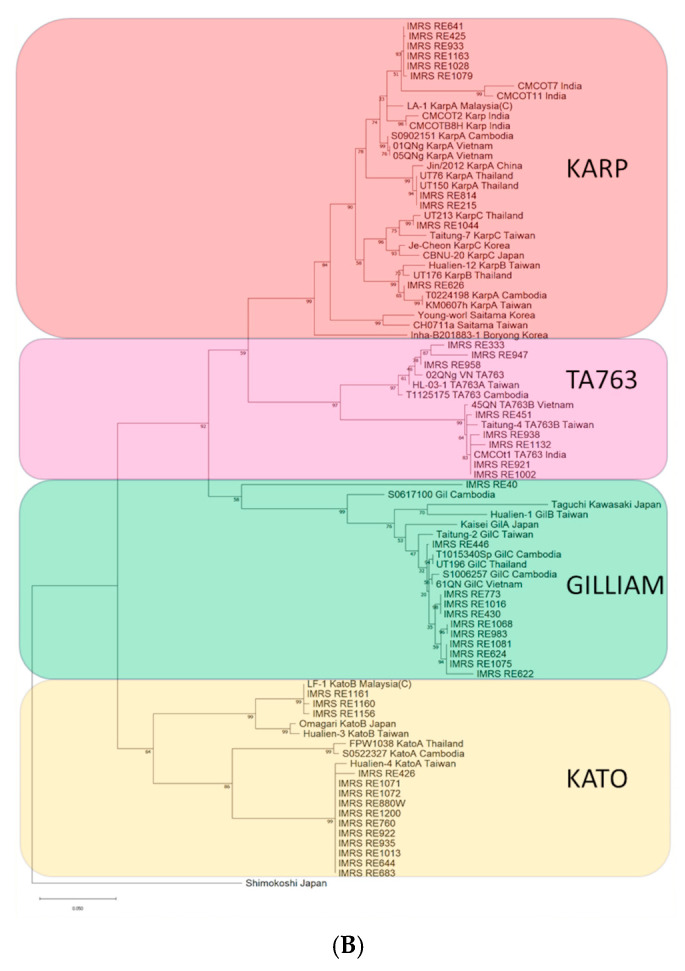
Phylogenetic analysis of the OT samples targeting *tsa56*. (**A**) The circular phylogenetic tree consists of 106 local isolates (nested and single-run PCR) and 50 isolates from other countries (Table S1) and is based on the 450 bp consensus region of *tsa56.* (**B**) The rectangular phylogenetic tree portrays the relationships between 43 local isolates of single-run PCR and those from other countries (consensus region of *tsa56,* 873 bp) (Table S1). The OT genotypes are illustrated by the line colour: Karp (red), Gilliam (green), TA763 (pink), and Kato (yellow). The tree is generated by the maximum likelihood method with 1000 bootstrap replicates using Mega 11.

A total of 106 *htr*A positive samples from qPCR were processed with nPCR and single-run PCR. The majority of the samples were of age group 31–40 years old, male, and of Malay ethnicity (Table 1). The Pahang state of Peninsular Malaysia contributed the majority of the acute scrub typhus cases at 52.8%, followed by Negeri Sembilan, Selangor, Kuala Lumpur, Kelantan, Sarawak, Kuala Lumpur, Melaka, Perak, and Johor at a decreasing trend. A total of 85% of the samples were extracted from the blood, followed by eschar, CSF, and urine (Table 1). OT genotyping was analyzed based on partial *tsa56* gene (Figure 1A) phylogenetic tree analysis compared with other known OT genotypes available in the public database (Table S1). In addition, phylogenetic analysis was also performed on 43 sequences generated from single-run PCR (Figure 1B). The results were similar with the first tree (Figure 1B vs. Figure 1A), suggesting that single-run PCR is suitable as an alternative for nPCR.

**Figure 2:**
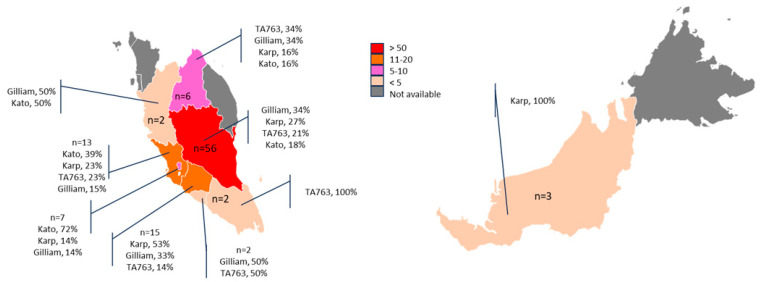
Distribution of OT genotypes in Malaysia from December 2022 to November 2024. The map includes the Peninsular Malaysia and northern Borneo (Sarawak and Sabah) states of Malaysia. A total of 106 OT genotypes are defined (Table S1). The number of cases are denominated by different colour: >50 cases (red), 11–20 cases (orange), 5–10 cases (pink), <5 cases (beige), and no cases available (grey).

Based on the phylogenetic tree analysis, Gilliam (*n* = 31, 29.2%) and Karp (*n* = 31, 29.2%) are the dominant genotypes in Malaysia followed by TA763 (*n* = 22, 20.8%) and Kato (*n* = 22, 20.8%). All four genotypes were observed in the Peninsular Malaysia whilst only the OT genotype Karp in Sarawak. (i) OT genotype Gilliam has the highest prevalence in Pahang, Perak, and Johor; (ii) OT genotype Karp in Negeri Sembilan and Sarawak; (iii) OT genotype Kato in Selangor and Kuala Lumpur; (iv) OT genotype TA763 and Gilliam in Kelantan (Figure 2).

**Figure 3:**
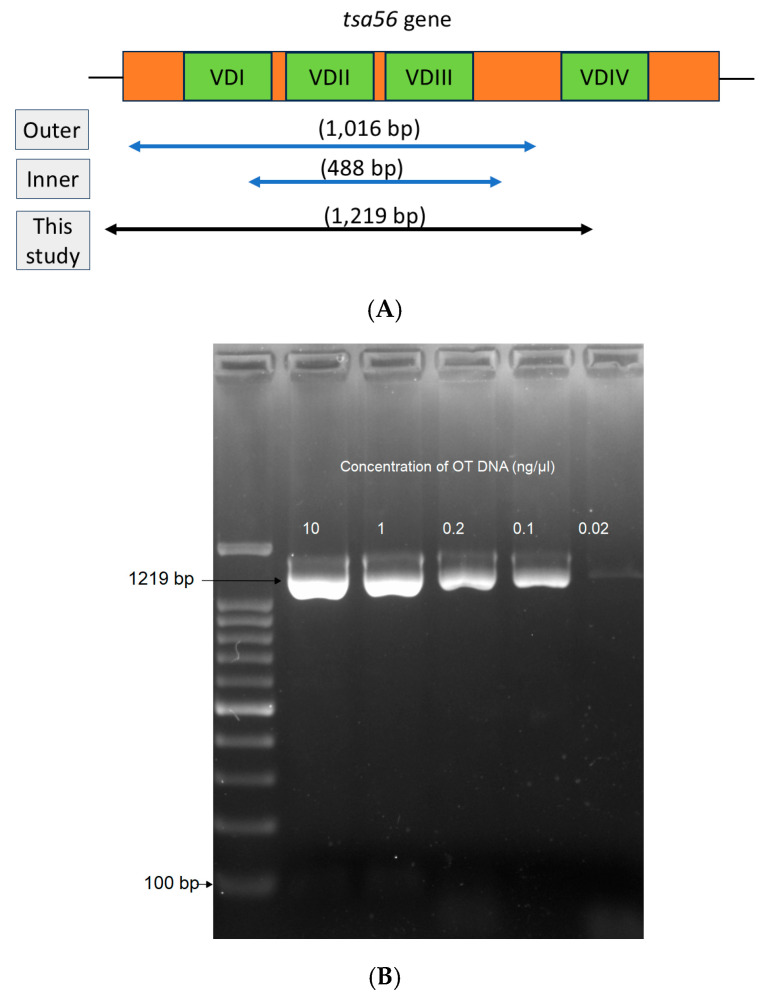
(**A**) The primers targeting the *tsa56* gene and hypervariable regions (VDI, VDII, VDIII, and VDIV) used in this study. The outer and inner fragments targeted by nested PCR (488 bp, blue arrow). The new primer for the single-run PCR proposed in this study (1219 bp, black arrow). (**B**) Gel image showing the detection limit of the single-run PCR. The OT DNA was tested from 0.02 ng/µL to 10 ng/µL, showing a single intact band at 1219 bp.

A random selection of DNA (*n* = 12) of a Ct value within the range of ≥28 to <38 and a positive nested PCR were used to evaluate the performance of the new single-run PCR protocol proposed in this study. This protocol is able to generate a long sequence of 1219 bp in a single-run PCR (Figure 3A,B), which takes approximately 70 min to complete. The presence of an intact 1219 bp band on a 1.5% agarose gel was interpreted as positive OT. The single-run PCR is able to detect as low as 0.02 ng/µL OT DNA (Figure 3B). A 5 µL DNA template from clinical samples is required for optimum performance. Genotype analysis could be concluded upon phylogenetic analysis on Sanger sequencing results. The 12 sequences of the single-run PCR were aligned with the respective nPCR products and showed 100% similarity (Figure S1C). The sequences have been deposited in GenBank (accession numbers: PV442421-PV442437).

## 4. Discussion

The findings of this study indicate that acute scrub typhus infection is prevalent and likely underreported in Malaysia. However, our capacity to estimate the national prevalence of OT was limited, as sample submissions were not received from all states and several positive cases lacked sufficient material for genotyping. Consequently, this report focuses on the genotypic characteristics of OT based on the available specimens (*n* = 106). Among these, the Gilliam and Karp groups were identified as the predominant circulating genotypes in Malaysia, consistent with observations from other endemic regions [9,12,18,19,20,21,22]. It should be noted that the conventional nested PCR assay, which targets a short region spanning VD I to VD III, may miss out new variations in the genotypes.

Overall, the results showed that Malaysian OT samples, including OTM1 and OTM3 of year 2013 [23], are region-specific and are clustered within the country’s own population. For instance, Malaysian OT samples are grouped together under the same branch, slightly diverging from other countries such as Cambodia, Vietnam, Thailand, India, Taiwan, and China. This phenomenon is also observed in other countries where the *tsa56* genes of the same genotypes portray higher similarity within the same country compared to others (Figure 1, [9,10,11]). In this study, we observed Gilliam and Karp as the dominant OT genotypes in Malaysia, which tallies with previous observations [23,24,25]. Surprisingly, Malaysian OT samples are more closely related to Bangladesh’s and India’s for Karp and Taiwan’s for Kato, whilst Gilliam’s and TA763’s are more related to neighbouring countries such as Vietnam, Cambodia, and Thailand (Figure 1). However, compared to neighbouring countries, the samples used in this study are distantly located from Japan and Korea. Despite being grouped within the same genotype, there are slight variations in *tsa56* sequences between different countries, postulating the importance of geographical distribution and the incorporation of a regions’ genotype for future diagnostic and vaccine developments. We also observed that the current circulating Karp and Kato genotypes in Malaysia branched together with LA-1 and LF-1 (isolated from Malaysian chiggers), respectively, which were isolated 30 years ago (Figure 1) [11], suggesting that there have been few recombination or mutation events occurring within the local population over the last three decades.

A further investigation into the OT genotypes from various states in Malaysia showed that there are different dominant OT genotypes in different states (Figure 3). Pahang state, which covers a large land area bordering with Kelantan, Perak, Selangor, and Negeri Sembilan, showed different proportion of OT genotypes. For instance, Kato is more commonly found in Selangor instead of Gilliam, which is predominant in Pahang. Nevertheless, in this study, Pahang contributes >50% of the scrub typhus cases. This observation could be due to the fact that agricultural and recreational activities are prominent in Pahang and that it has a large area of rainforest. However, 19% of the cases were from Kuala Lumpur and Selangor, which are the centre economic hubs of Malaysia. Given the ease of transportation and deforestation process disrupting the habitat of the vectors, scrub typhus may spread from rural to urban area [26]. This is supported by the clustering of the OT genotypes regardless of the states. For instance, within Karp, the samples from Pahang, Selangor, Sarawak, Kuala Lumpur, and Negeri Sembilan were clustered together (Figure 1A, Table S1).

Kumaraswany et al. reported the wrong grouping of Karp andTA763 based on nested PCR for CMCOT7 and CMCOT11 [10]. In this study, we observed a slight diverge of these two isolates, but they were still within the Karp group using both nPCR and single-run PCR. Further analysis on Karp and TA763 genotypes showed that there is low percent identity match between these two isolates with both genotypes (<90%). Instead, CMCOt1 (TA763, India) exhibited 89% similarity with CMCOT7 (Figure S1B). In addition, six of our local OT samples (IMRS_RE641, IMRS_RE425, IMRS_RE933, IMRS_RE1163, IMRS_RE1028, and IMRS_RE1079) shared the same node as CMCOT7 and CMCOT11 and showed >90% similarity with the Karp genotype, compared to 70–85% with the TA763 genotype (Figure S1B).

Serological testing to detect antibodies may not be sufficient in scrub typhus endemic areas, making diagnostics a difficult task. The confirmation of scrub typhus requires an increase of four-fold titers between two consecutive samples [27]. This led to confusion when the second sample is not available. Therefore, molecular detection by PCR is crucial, especially for the early detection of scrub typhus. It is worth noting that due to OT being an obligate intracellular bacteria, conventional culture methods cannot be performed and the yield in clinical samples is low. Hence, a high-fidelity PCR protocol is important to increase the sensitivity of the assay. Our proposed single-run PCR protocol showed similar results with traditionally used nPCR. Therefore, single-run PCR, which requires <2 h to complete, is a good candidate as an alternative to nPCR, which requires 4–5 h in total.

To the best of our knowledge, despite the low sample size, our current study provides the first whole picture illustrating the circulating OT genotypes among human cases in Malaysia, apart from the sporadic cases in human and chiggers reported earlier [23,24,25]. Our data suggests that apart from Gilliam and Karp, TA763, which is the predominant genotype in India, is on the rise in Malaysia. The limitations in this study are (i) the information on the OT incidence data, as we did not receive samples from some state hospitals in Malaysia; (ii) the low DNA load from clinical specimen and the availability of the samples to incorporate into our proposed single run protocol; (iii) the sequencing fidelity of Sanger sequencing as it is able to sequence 800–1000 bp of the single-run PCR protocol, but the length is sufficient for genotypic analysis.

It is to note that the DNA load in clinical specimen may vary between different sample types, genotypes, highest body temperature during fever onset, ethnic group, vector type, liver enzymes (AST, ALT, AP), and total bilirubin level in blood [20,28]. Karp genotype was reported to correlate with higher DNA loads, severe characteristics, and slower treatment response in patients [28,29]. Our study warrants a further investigation into the association of the genotypes and disease severity for future work.

## 5. Conclusions

Gilliam and Karp are the predominant circulating OT genotypes in Malaysia, followed by TA763 and Kato. Pahang state of Malaysia is a hot-spot for acute scrub typhus and scrub typhus should be included in the differential diagnosis of acute febrile illness. The single-run PCR is time-saving and a promising approach for OT detection and genotype analysis in one run to complement a clinical diagnostic setting.

## Figures and Tables

**Table 1:** Demographic and clinical features and sample type of patients with positive OT DNA used in this study.

**Demography**	***n* = 106 (%)**
*i. Age group*	
0–10	1 (0.9)
11–20	7 (6.6)
21–30	17 (16.1)
31–40	24 (22.7)
41–50	19 (17.9)
51–60	23 (21.7)
>60	15 (14.1)
*ii. Gender*	
Male	77 (72.6)
Female	29 (27.4)
*iii. Ethnicity*	
Malay	65 (61.3)
Chinese	9 (8.4)
Aborigine *	6 (5.7)
Indian	6 (5.7)
Foreigner	20 (18.9)
*iv. Residing state*	
Pahang	56 (52.8)
Negeri Sembilan	15 (14.1)
Selangor	13 (12.3)
Kuala Lumpur	7 (6.6)
Kelantan	6 (5.7)
Sarawak	3 (2.8)
Johor	2 (1.9)
Melaka	2 (1.9)
Perak	2 (1.9)
**Clinical features**	***n* = 106 (%)**
Fever Vomiting	99 (93)
Headache	42(40)
Eschar	42 (40)
Nause	32 (30)
Diarrhea	17 (16)
Rash	12 (11)
Myalgia	11 (10)
Lethargy	11 (10)
Lymphadenopathy	11 (10)
Cough	8 (8)
Poor appetite	7 (7)
Dizziness	6 (6)
Abdominal pain	5 (5)
**Sample type**	***n* = 106 (%)**
Blood	90 (85)
Eschar	14 (13.2)
CSF	1 (0.9)
Urine	1 (0.9)

* Aborigine ethnicity refers to Orang Asli in Peninsular Malaysia or Bumiputra Sarawak.

## Data Availability

The original contributions presented in this study are included in the article/Supplementary Material. Further inquiries can be directed to the corresponding author.

## Supplementary Materials

The following supporting information can be downloaded at: https://www.mdpi.com/article/10.3390/tropicalmed10090252/s1, Figure S1: Multiple sequence alignment. Table S1: List of 156 *tsa56* genes and their information used in this study.
