# Supplementary material for: Genotype Analysis on Orientia tsutsugamushi Causing Scrub Typhus in Malaysia: A Re-Emerging Disease
Source: Trop Med Infect Dis. 2025 Sep 2;10(9):252. doi: 10.3390/tropicalmed10090252 (PMC12474467; doi:10.3390/tropicalmed10090252)
Supplement: Supplementary file 1 [file tropicalmed-10-00252-s001.zip › 3785082_Figure S1.pdf]

A.

```
CLUSTAL O(1.2.4) multiple sequence alignment


Jin/2012         MKKIMLIASAMSALSLPFSASAIELGDEGGLECGPYAKVGVVGGIITGVESARLDPADTD         60
IMRS_RE215       --KIMLIASAMSALSLPFSASAIELGDEGGLECGPYAKVGVVGGMITGVESIRLDPADAD         58
UT150            MKKIMLIASAMSALSLPFSASAIELGDEGGLECGPYAKVGVVGGMITGVESIRLDPADAD         60
IMRS_RE814       -------------LSLPFSASAIELGDEGGLECGPYAKVGVVGGMITGVESIRLDPADAD         47
Taitung-7        MKKIMLIASAMSALSLPFSASAIELEAEGGLECGPYAKVGVVGGMITGVESTRLDPADAD         60
UT213            MKKIMLIASAMSALSLPFSASAIELGDEGGLECGPYAKVGVVGGMITGVESTRLDLADAD         60
IMRS_RE1044      -----------SALSLPFSASAIELGDEGGLECGPYAKVGVVGGMITGVESTRLDPADAD         49
Je-cheon         MKKIMLIASAMSALSLPFSASAIELEDEGGLECGPYAKVGVFGGMITGVESTRLDPADAD         60
CBNU-20          MKKIMLIASAMSALSLPFSASAIELDDEGGLECGPYAKVGVVGGMITGVESARLDPADVD         60
T0224198         MKKIMLIASAMSALSLPFSASAIELGDEGGLECGPYGKVGIVGGMITGAESTRLDSSDAE         60
UT176            MKKIMLIASAMSALSLPFSASAIELGDEGGLECGPYGKVGIVGGMITGVESTHLDSADPE         60
Hualien-12       MKKIMLIASAMSALSLPFSASAIELGDEGGLECGPHAKVGIVGGIITGVESTRLDPADTD         60
IMRS_RE626       -KKIMLIASAMSALSLPFSASAIELGDEGGLECGPYGKVGIVGGMITSVESTRLDPADTD         59
IMRS_RE425       ---IMLIASAMSALSLPLSASAIELGEEGGLECGPYGKVGIVGGMITGVESIHLDSADTG         57
IMRS_RE1079      --KIMLIASAMSALSLPLSASAIELGEEGGLECGPYGKVGIVGGMITGVESIHLDSADTG         58
IMRS_RE1163      -----------SALSLPLSASAIELGEEGGLECGPYGKVGIVGGMITGVESIHLDSADTG         49
IMRS_RE1028      MKKIMLIASAMSALSLPLSASAIELGEEGGLECGPYGKVGIVGGMITGVESIHLDSADTG         60
IMRS_RE641       -KKIMLIASAMSALSLPLSASAIELGEEGGLECGPYGKVGIVGGMITGVESIHLDSADTG         59
IMRS_RE933       --KIMLIASAMSALSLPLSASAIELGEEGGLECGPYGKVGIVGGMITGVESIHLDSADTG         58
LA-1             MKKIMLIASAMSALSLPFSASAIELGDEGGLECGPYAKVGVVGGMITGVESARLDPADHE         60
CMCOT2           MKKIMLIASAMSALSLPFSASAIELGDEGGLECGPYAKVGVVGGMITGVESARLDPADAD         60
05QNg            MKKIMLIASARSALSLPFSASAIELGDEGGLECGPYGKVGIVGGMITSVESTRLDPADTD         60
S0902151         MKKIMLIASAMSALSLPFSASAIELGDEGGLECGPYGKVGIVGGMITSVESTRLDPADTD         60
01QNg            MKKIMLIASAMSALSLPFSASAIELGDEGGLECGPYGKVGIVGGMITSVESTRLDPADTD         60
T1125175         ---------------------------------------------LTGVESARLDPADHE         15
02QNg            MKKIMLIASAMSALSLPFSASAIELGDEGGLECGPYAKVGVVGGMITGVESARLDPADTG         60
Taitung-4        MKKIMLIASAMSALSLPFSASAIELGDEGGLECGPYAKVGIVGGMITGAESTRLDSSDAE         60
IMRS_RE938       ----------------------IELGDEGGLECGPYAKVGIVGGMITGAESTRLDSSDAE         38
IMRS_RE1132      MKKIMLIASAMSALSLPFSASAIELGDEGGLECGPYAKVGIVGGMITGAESTRLDSSDAE         60
CMCOt1           MKKIMLIASAMSALSLPFSASAIELGDEGGLECGPYAKVGIVGGMITGAESTRLHSSDAE         60
IMRS_RE921       ---IMLIASAMSALSLPFSASAIELGDEGGLECGPYAKVGIVGGMITGAESTRLDSSDAE         57
CMCOT7           MKKIMLIASAMSALSLPFSASAIELGDEGGLECGPYAKVGVVGGMITGVESARLDPADAD         60
CMCOT11          MKKIMLIASAMSALSLPFSASAIELGDEGGLECGPYAKVGVVGGMITGVESARLDPADAD         60
                                                              :*..** :*. :*


Jin/2012         GKKLLPLTTSMPFGGTLAAGMTIAQGFRAELGVMYLTNITA----QVEEGKVKADSGGKT         116
IMRS_RE215       GKKHLPLITSMPFGGTLAAGMTIAQGFRAELGVMYLTNITA----QVEEGKVKADSGGKT         114
UT150            GKKHLPLITSMPFGGTLAAGMTIAQGFRAELGVMYLTNITA----QVEEGKVKADSGGKT         116
IMRS_RE814       GKKHLPLITSMPFGGTLAAGMTIAQGFRAELGVMYLTNITA----QVEEGKVKADSGGKT         103
Taitung-7        GKKYLSLTTGIPFGGTLAAGMTIAPGFRAELGVMYLTNITA----QVEEGKVK-------         109
UT213            GKKHLSLTTGIPFGGTLAAGMTIAPGFRAELGVMYLTNITAQVTAQVEEGKGK-------         113
IMRS_RE1044      GKKHLSLTTGIPFGGTLAAGMTIAPGFRAELGVMYLTNITA----QVEEGKGK-------         98
Je-cheon         GKKHLSLTTGLPFGGALAAGMTIAPGFRAELGVMYLTNITA----QVEEGKVK-------         109
CBNU-20          GKKHLSLTSGLPFGGTLAAGMTIAPGFRAELGVMYLTNITA----QVEEGKVK-------         109
T0224198         GKKRLSLTTSMPFGGTLAAGMTIAQGFRAELGVMYLTNITA----QVEEGKVK-------         109
UT176            GKKRLPLTTSMPFGGTLAAGMTIAQGFRAELGIMYLTNITA----QVEEGKVK-------         109
Hualien-12       GKKLLPLTTSMPFGGTLAAGMTIAQGFRAELGVMYLTNITA----QVEEGKVK-------         109
IMRS_RE626       GKKHLPLTTSMPFGGTLAAGMTIAPGFRAELGVMYLTNITA----QVEEGKVK-------         108
IMRS_RE425       GKKHLPLTTSMPFGGTLTAGMTIAPGFRAELGVMYLTNITA----QVEEGKVK-------         106
IMRS_RE1079      GKKHLPLTTSMPFGGTLAAGMTIAPGFRAELGVMYLTNITA----QVEEGKVK-------         107
IMRS_RE1163      GKKHLPLTTSMPFGGTLAAGMTIAPGFRAELGVMYLTNITA----QVEEGKVK-------         98
IMRS_RE1028      GKKHLPLTTSMPFGGTLAAGMTIAPGFRAELGVMYLTNITA----QVEEGKVK-------         109
IMRS_RE641       GKKHLPLTTSMPFGGTLAAGMTIAPGFRAELGVMYLTNITA----QVEEGKVK-------         108
IMRS_RE933       GKKHLPLTTSMPFGGTLAAGMTIAPGFRAELGVMYLTNITA----QVEEGKVK-------         107
LA-1             GKKNLPLTTSMPFGGTLAAGMTIAQGFRAELGVMYLTNITA----QVEEGKVK-------         109
CMCOT2           GKKQLPLTTSMPFGGTLAAGMTIAPGFRAELGVMYLTNITA----QVEEGKVK-------         109
05QNg            GKKHLLLTTSMPFGGTLAAGMTIAPGFRAELGVMYLTNITA----QVEEGKVK-------         109
S0902151         GKKHLPLTTSMPFGGTLAAGMTIAPGFRAELGVMYLTNITA----QVEEGKVK-------         109
01QNg            GKKHLLLTTSMPFGGTLAAGMTIAPGFRAELGVMYLTNITA----QVEEGKVK-------         109
T1125175         GKKHLPLTTSMPFGGTLAAGMTIAQGFRAELGVMYLTNITA----QVEEGKVKADSGGKT         71
02QNg            GKKQLPLTTSMPFGGTLAAGMTIASGFRAEIGVMYLRNISA----EVELGKVKADSGSKT         116
Taitung-4        GKKRLSLTTSVPFGGTLAAGITIAQGFRAELGVMYLTNITA----QVEEGKGKIGSDVAK         116
IMRS_RE938       GKKRWSLTTSMPFGGTLAAGMTIAQGFRAELGVMYLTNITA----QVEEGKVKIGCNVAK         94
IMRS_RE1132      GKKRLSLTTSMPFGGTLAAGMTIAQGFRAELGAMYLTNITA----QVEEGKGKIRSNAAK         116
CMCOt1           GKKRLSLTTSMPFGGTLAAGMTIAQGFRAELGVMYLTNITA----QVEEGKGKIGSNVAK         116
IMRS_RE921       GKKRLSLTTSMPFGGTLAAGMTIAQGFRAELGVMYLTNITA----QVEEGKGKIGSNVAK         113
CMCOT7           GKKQLPLTTSMPFGGTLAAGMTIAPGFRAELGVMYLTNITA----QVEEGKVKADSNVDK         116
CMCOT11          GKKQLPLTTSMPFGGTLAAGMTIAPGFRAELGVMYLTNITA----QVEEGKVKADSEVDK         116
                 ***   * :.:****:*:**:*** *****:* *** **:*    :** ** *
```

```
Jin/2012            KADSGGEIKADSGGGTDVPIRKRFKLTPPQPTIMPISIADRDFGIDIRNIPQAQAG----   172
IMRS_RE215          KADSGGEIKADSGGGTDAPIRKRFKLTPPQPTIMPISIADRDFGIDIRNIPQAQAG----   170
UT150               KADSGGEIKADSGGGTDAPIRKRFKLTPPQPTIMPISIADRDFGIDIRNIPQAQAG----   172
IMRS_RE814          KADSGGEIKADSGGGTDAPIRKRFKLTPPQPTIMPISIADRDFGIDIRNIPQAQAG----   159
Taitung-7           -ADSVGETKADSVGGKDSSIRKRFKLTPPQPTIMPISIADRDFGIDIPNIPQQQAQAAQP   168
UT213               -VDSRGEIKADSGGGTDAPIRKRFKLTPPQPTISLISIADRDFGIDIPNIPQQQAQAAQP   172
IMRS_RE1044         -VDSRGEIKADSGGGTDAPIRKRFKLTPPQPTIMPISIADRDFGIDIPNIPQQQAQAAQP   157
Je-cheon            -ADSIGETKADSVGGKDAPIRKRFKLTPPQPTIMPISIADRDFGIDIPNIPQQQAQAAQP   168
CBNU-20             -TDSIGEAKADSVGGKDASIRKRFKLTPPQPTIMPISIADRDFGIDIPNIPQQQAQAAQP   168
T0224198            -ADSGGKTKADSGGGTDAPIRKRFKLTPPQPTIMPISIADRDFGIDIPNIRQAQARAAQP   168
UT176               -ADSGGKTKADSGGGTDAPIRKRFKLTPPQPTIMPISIADRDFGIDIPNIRQAQARAAQP   168
Hualien-12          -ADSGGKTKADSGGGTDAPIRKRFKLTPPQPTIMPISIADRDFGIDIPNIRQAQARAAQP   168
IMRS_RE626          -ADSGGKTKADSGGGTDAPIRKRFKLTPPQPTIMPISIADRDFGIDIPNIRQAQARAAQP   167
IMRS_RE425          -ADSGGKTKADSGGGTDAPIRKRFKLTPPQPTIMPISIADRDFGIDICNIPQQQAQAANP   165
IMRS_RE1079         -ADSGGKTKADSGGGTDAPIRKRFKLTPPQPTIMPISIADRDFGIDICNIPQQQAQAANP   166
IMRS_RE1163         -ADSGGKTKADSGGGTDAPIRKRFKLTPPQPTIMPISIADRDFGIDICNIPQQQAQAANP   157
IMRS_RE1028         -ADSGGKTKADSGGGTDAPIRKRFKLTPPQPTIMPISIADRDFGIDICNIPQQQAQAANP   168
IMRS_RE641          -ADSGGKTKADSGGGTDAPIRKRFKLTPPQPTIMPISIADRDFGIDICNIPQQQAQAANP   167
IMRS_RE933          -ADSGGKTKADSGGGTDAPIRKRFKLTPPQPTIMPISIADRDFGIDICNIPQQQAQAANP   166
LA-1                -ADSGGKTKADSGGETDAPIRKRFKLTPPQPTIMPISIADRDFGIDICNIPQAQAQAANP   168
CMCOT2              -ADSGGKTKADSGGGTDAPIRKRFKLTPPQPTIMPISIADRDFGIDICNIPHAQAQAANP   168
05QNg               -ADSGGKTKADSGGGTDAPIRKRFKLTPPQPTIMPISIADRDFGIDIPNIPQAQAQAANP   168
S0902151            -ADSGGKTKADSGGGTDAPIRKRFKLTPPQPTIMPISIADRDFGIDIPNIPQAQAQAANP   168
01QNg               -ADSGGKTKADSGGGTDAPIRKRFKLTPPQPTIMPISIADRDFGIDIPNIPQAQAQAANP   168
T1125175            KADSGGE--------TDAPIRKRFKLTPPQPTIMPISIADRDFGVDVTNIPQAQVQPPQQ   123
02QNg               KADSGGE--------TDAPIRKRFKLTPPQPTIMPISIADRDFGVDVTNIPQAQVQPPQQ   168
Taitung-4           GSDAANDNTG-TDANQAKQLKRPPKLTPPQPVIMPISTADRDMGVDVINEPQAQVPQ-AQ   174
IMRS_RE938          GSDAANDITG-TDANQAKQLKRPPKLTPPQPVIMPISTADRDMGVDVINEPQAQVAQ-AQ   152
IMRS_RE1132         GSDAANDNTG-TDANQAKHLKPPPKLTPPQPVIMPISTADRDMGVDVINEPQAQVAQ-AQ   174
CMCOt1              GSDAANDNTG-TDANQAKQLKRPPKLTPPQPVIMPISTADRDMGVDVINEPQAQVAQ-AQ   174
IMRS_RE921          GSDAANDNTG-TDANQAKQLKRPPKLTPPQPVIMPISTADRDMGVDVINEPQAQVAQ-AQ   171
CMCOT7              GSDSANDNTG-TDANQAKQRKRPPKLTPPQPTIMPISIADRDLGVDICNIPQAQVAQ-AQ   174
CMCOT11             GSDSGKDKTG-TDAXXAILRKRLFKLTPPQPTIMPISIADRDLGIDICNIPQAQLAQ-AP   174
                      *:  .             :   *******.*  ** ****:*:*: *  : *

Jin/2012            NLNNEQRAAARIAWLKNCAGIDYRVKNPNNPNGPMVINPILLNIPQGNPIPVG----NQR   228
IMRS_RE215          NLNNEQRAAARIAWLKNCAGIDYRVKDPNNPNGPMVINPILLNIPQGNPIPVG----NQR   226
UT150               NLNNEQRAAARIAWLKNCAGIDYRVKDPNNPNGPMVINPILLNIPQGNPIPVG----NQR   228
IMRS_RE814          NLNNEQRAAARIAWLKNCAGIDYRVKDPNNPNGPMVINPILLNIPQGNPIPVG----NQR   215
Taitung-7           QLNDEQRAAARITWLKNCAGIDYRVKNPNDPNGPMVINPILLNIPQGNPNPV--GNPPQR   226
UT213               QLNDEQRAAARIAWLKNCAGIDYRVKNPNDPNGPMVINPILLNIPQGNPNPV--GNPPQR   230
IMRS_RE1044         QLNDEQRAAARIAWLKNCAGIDYRVKNPNDPNGPMVINPILLNIPQGNPNPV--GNPPQR   215
Je-cheon            QLNDEQRAAARIAWLKNCAGIDYRVKNPNDPNGPMVINPILLNIPQGNPNPA--GNPPQR   226
CBNU-20             QLNDEQRAAARITWLKNCAGVDYRVKNPNDPNGPMVINPILLNIPQGNPNPV--GNPPQR   226
T0224198            PLNDEQRAAARIAWLKNYAGIDYRVKNPNDPNGPMVINPILLNIPQGDPHPAAGQQPPQR   228
UT176               PLNDEQRAAARIAWLKNYAGIDYRVKNPNDPNGPMVINPILLNIPQGDPHPA--GQPPQR   226
Hualien-12          PLNDEQRAAARIAWLRIYAGIDYRVKNPNDPNGPMVINPILLNIPQGDPHPA--GQPPQR   226
IMRS_RE626          PLNDEQRAAARIAWLKNYAGIDYRVKNPNDPNGPMVINPILLNIPQGDPHPA--GQPPQR   225
IMRS_RE425          ALNDEQRAAARIAWLKNCAGIDYRVKDPNNPNGPMVINPILLNIPQGNPNPA--GNPPQR   223
IMRS_RE1079         ALNDEQRAAARIAWLKNCAGIDYRVKDPNNPNGPMVINPILLNIPQGNPNPA--GNTPQR   224
IMRS_RE1163         ALNDEQRAAARIAWLKNCAGIDYRVKDPNNPNGPMVINPILLNIPQGNPNPA--GNPPQR   215
IMRS_RE1028         ALNDEQRAAARIAWLKNCAGIDYRVKDPNNPNGPMVINPILLNIPQGNPNPA--GNPPQR   226
IMRS_RE641          ALNDEQRAAARIAWLKNCAGIDYRVKDPNNPNGPMVINPILLNIPQGNPNPA--GNPPQR   225
IMRS_RE933          ALNDEQRAAARIAWLKNCAGIDYRVKDPNNPNGPMVINPILLNIPQGNPNPA--GNPPQR   224
LA-1                ALNDEQRAAARIAWLKNCAGIDYRVKDPNNPNGPMVINPILLNIPQGNPNPA--GNPPQR   226
CMCOT2              ALNDEQRAAARIAWLKNCAGIDYRVKDPNNPNGPMVINPILLNIPQGNPNPA--GNPPQR   226
05QNg               QLNDEQRAAARIAWLKNCAGIDYRVKDPNNPNGPMVINPILLNIPQGNPNPA--GNPPQR   226
S0902151            QLNDEQRAAARIAWLKNCAGIDYRVKDPNNPNGPMVINPILLNIPQGNPNPA--GNPPQR   226
01QNg               QLNDEQRAAARIAWLKNCAGIDYRVKDPNNPNGPMVINPILLNIPQGNPNPA--GNPPQR   226
T1125175            ANDPLVRGVRRIAWLKEYAGIDYMVKDPNNP-GRMMVNPVLLNIPQGPPAQN----P---   175
02QNg               ANDPLVRGVRRIAWLKEYAGIDYMVKDPNNP-GRMMVNPVLLNIPQGPPAQN----P---   220
Taitung-4           QNDPLVRGLRRIAWLKQYAGIDYMVKDPNNP-GQMMVNPVLLNIPQGPPANN----P---   226
IMRS_RE938          QNDPLVRGLRRIAWLKQYAGIDYMVKDPNNP-GQMMVNPVLLNIPQGPPANN----P---   204
IMRS_RE1132         QNDPLVRGLRRIAWLKQYAGIDYMVKDPNNP-GQMMVNPVLLNIPQGPPANN----P---   226
CMCOt1              QNDPLVRGLRRIAWLKQYAGIDYMVKDPNNP-GQMMVNPVLLNIPQGPPANN----P---   226
IMRS_RE921          QNDPLVRGLRRIAWLKQYAGIDYMVKDPNNP-GQMMVNPVLLNIPQGPPANN----P---   223
CMCOT7              QNDPLVRASPRIAWLKNCAGIDYRVKDPNNP-GPMVINPILLNIPQGPPAGN----P---   226
CMCOT11             HNDPLVRAAARIAWLKNCAGIDYRVKDPNNP-GPMVINPILLNIPQGPPAGN----P---   226
                      :   *.  **:**:  **:** **:**:* * *::**:******* *
```

```
Jin/2012        AQQPAAFAIHDHEQWRYLVTGLAALSNANKPSASPVKVLSDKITQIYSDIKLFADIAGID    288
IMRS_RE215      AQQPAGFAIHDHEQWRYLVTGLAALSNANKPSASPVKVLSDKITQIYSDIKLFADIAGID    286
UT150           AQQPAGFAIHDHEQWRYLVTGLAALSNANKPSASPVKVLSDKITQIYSDIKLFADIAGID    288
IMRS_RE814      AQQPAGFAIHDHEQWRYLVTGLAALSNANKPSASPVKVLSDKITQIYSDIKLFADIAGID    275
Taitung-7       ANPPAGFAIHNHEQWRHLVVGLAALSNANKPSASPVKVLSDKITQIYSDIKPFADIAGIN    286
UT213           ANPPAGFAIHNHEQWRHLVVGLAALSNANKPSASPVKVLSDKISQIYSDIKPFADIAGID    290
IMRS_RE1044     ANPPAGFAIHNHEQWRHLVVGLAALSNANKPSASPVKVLSDKISQIYSDIKPFADIAGID    275
Je-cheon        ANPPAGFAIHNHEQWRHLVVGLAALSNANKHSAPPVKVLSDKITQIYSDIKPFADIAGID    286
CBNU-20         ANPPAGFAIHNHEQWRNLVVGLAALSNANKPSASPVKVLSDKITQIYSDIKPFADIAGID    286
T0224198        ANPPEDFEIHDHEQWRYLVVGLAALSNANKPSASPVKVLSDKITQIYSDVKLFADIAGID    288
UT176           ANPPDDFEIHDHEQWRHLVVGLAALSNANKPSASPVKVLSDKITQIYSDVKPFADIAGID    286
Hualien-12      ANPPEDFEIHDHEQWRHLVVGLAVLSNANKPSASPVKVLSDKITQIYSDIKPFADIAGID    286
IMRS_RE626      ANPPEDFEIHDHEQWRHLVVGLAALSNANKPSASPVKVLSDKITQIYSDVKLFADIAGID    285
IMRS_RE425      AQQPANFAIHNHDQWRHLVVGLAALSNANKPSASPVKVLSDKITQIYSDIKPFADIAGID    283
IMRS_RE1079     AQQPANFAIHNHDQWRHLVVGLAALSNANKPSASPVKVLSDKITQIYSDIKPFADIAGID    284
IMRS_RE1163     AQQPANFAIHNHDQWRHLVVGLAALSNANKPSASPVKVLSDKITQIYSDIKPFADIAGID    275
IMRS_RE1028     AQQPANFAIHNHDQWRHLVVGLAALSNANKPSASPVKVLSDKITQIYSDIKPFADIAGID    286
IMRS_RE641      AQQPANFAIHNHDQWRHLVVGLAALSNANKPSASPVKVLSDKITQIYSDIKPFADIAGID    285
IMRS_RE933      AQQPANFAIHNHDQWRHLVVGLAALSNANKPSASPVKVLSDKITQIYSDIKPFADIAGID    284
LA-1            AQQPANFAIHNHDQWRHLVVGLAALSNANKPSASPVKVLSDKITQIYSDIKHLADIAGID    286
CMCOT2          AQQPANFAIHNHDHCRHLVVGLAALSNANKPSASPVKVLSDKITQIYSDIKPFADIAGID    286
05QNg           AQQPANFAIHNHDQWRHLVVGLAALSNANKPSASPVKVLSDKITQIYSDIKPFADIAGID    286
S0902151        AQQPANFAIHNHDQWRHLVVGLAALSNANKPSASPVKVLSDKITQIYSDIKPFADIAGID    286
01QNg           AQQPANFAIHNHDQWRHLVVGLAALSNANKPSASPVKVLSDKITQIYSDIKPFADIAGID    286
T1125175        RAAMQPCNILDHDHWRHLVVGVTALSNANKPSASPVKILSEKITQIYSDIRPFADIAGIN    235
02QNg           RAAMQPCNILDHDHWRHLVVGVTALSNANKPSASPVKILSEKITQIYSDIRPFADIAGIN    280
Taitung-4       RAPMQRCDILNHDHWRHLVVGIAALSNANKPSASPVKVLSDKITKIYSDIKPFADIAGID    286
IMRS_RE938      RAPMQRCDILNHDHWRHLVVGIAALSNANKPSASPVKVLSDKITQIYSDIKPFGDIAGID    264
IMRS_RE1132     RAPMQRCDILNHDHWRHLVVGIAALSNANKPSASPVKVLSDKITQIYSDIKPFADIAGID    286
CMCOt1          RAPMQRCDILNHDHWRHLVVGIAALSNANKPSASPVKVLSDKITQIYSDIKPFADIAGID    286
IMRS_RE921      RAPMQRCDILNHDHWRHLVVGIAALSNANKPSASPVKVLSDKITQIYSDIKPFADIAGID    283
CMCOT7          RAPMQXFAIHNHDHWRHLVVGLAALSNANKPSASPVKVLSDKITQIYSDIKPFADIAGID    286
CMCOT11         RAPMQIFAIHNHDHWRHLVVGLAALSNANKPSASPVKVLSDKITQIYSDIKPFADIAGID    286
                        * :*:: * **.*::.****** ** ***:**:**::****:: :.*****:


Jin/2012        VPDAGLPNSATVEQIQNKMQELNNVLEELRESFEGYLGGNAFANQIQLNFVMPQQAQQQQ    348
IMRS_RE215      VPDAGLPNSATVEQIQNKIQELNDVLEELRESFDGYLGGNAFANQIQLNFVMPQQAQ-QQ    345
UT150           VPDAGLPNSATVEQIQNKIQELNDVLEELRESFDGYLGGNAFANQIQLNFVMPQQAQ-QQ    347
IMRS_RE814      VPDAGLPNSATVEQIQNKIQELNDVLEELRESFDGYLGGNAFANQIQLNFVMPQQAQ-QQ    334
Taitung-7       VPDTGLPNSASVEQIQSKMQELNELLEELRDSFDGYIS-NAFANPIQLNFVMPQQAQQQQ    345
UT213           VPDTGLPNSASVEQIQSKMQELNDVLEELRDSFDGYIS-NAFVNQIQLNFVMPQQAQ-QQ    348
IMRS_RE1044     VPDTGLPNSASVEQIQSKMQELNDVLEELRDSFDGYIS-NAFVNQIQLNFVMPQQAQ-QQ    333
Je-cheon        VPDTGLPNSASVEQIQNKIQELSNVLEDLRDSFDGYIG-NAFANQIQLNFVMPQQAQQQQ    345
CBNU-20         VPDTGLPNSASVEQIQNKIHELSNVLEDLRDSFDGYIG-NAFANQIQLNFVMPQQAQQQQ    345
T0224198        VPDAGLPNSATVEQIQNKIQELNDLLEGLRESFEGYLGGNAFANQIQLNFVMPQQAQQQQ    348
UT176           VPDTSLPNSASVEQIQNKMQELNDLLEELRESFDGYLGGNAFANQIQLNFVMPQQAQ-QQ    345
Hualien-12      VPDTGLPNSASIEQIQNKMQELGDLLEELRESFDGYLGGNAFANQIQLNFVMPQQAQQQQ    346
IMRS_RE626      VPDAGLPNSATVEQIQNKMQELNDVLEELRESFDGYLGGNAFANQIQLNFVMPQQAQQQQ    345
IMRS_RE425      VPDTSLPNSASVEQIQNKMQELNDLLEELRESFDGYLGGNAFANQIQLNFVMPQQAQQQQ    343
IMRS_RE1079     VPDTSLPNSASVEQIQNKMQELNDLLEELRESFDGYLGGNAFANQIQLNFVMPQQAQQQQ    344
IMRS_RE1163     VPDTSLPNSASVEQIQNKMQELNDLLEELRESFDGYLGGNAFANQIQLNFVMPQQAQQQQ    335
IMRS_RE1028     VPDTSLPNSASVEQIQNKMQELNDLLEELRESFDGYLGGNAFANQIQLNFVMPQQAQQQQ    346
IMRS_RE641      VPDTSLPNSASVEQIQNKMQELNDLLEELRESFDGYLGGNAFANQIQLNFVMPQQAQQQQ    345
IMRS_RE933      VPDTSLPNSASVEQIQNKMQELNDLLEELRESFDGYLGGNAFANQIQLNFVMPQQAQQQQ    344
LA-1            VPDTSLPNSASVEQIQNKMQELNDVLEELRDSFDGYIS-NAFANQIQLNFVMPQQAQQQQ    345
CMCOT2          VPDTGLPNSASVEQIQRKMQELNDVLEGLRDAFDGYIN-NAFVDQIQLNFVMPPQAQQQQ    345
05QNg           VPDTSLPNSASVEQIQNKMQELNDVLEELRDSFDGYIS-NAFANQIQLNFVMPQQAQ-QQ    344
S0902151        VPDTSLPNSASVEQIQNKMQELNDVLEELRDSFDGYIS-NAFANQIQLNFVMPQQAQ-QQ    344
01QNg           VPDTSLPNSASVEQIQNKMQELNDVLEELRDSFDGYIS-NAFANQIQLNFVMPQQAQ-QQ    344
T1125175        VPDTGLPNSASVEQIQSKMQELSDLLEELRDSFDGYIS-NAFAGQIQLNFVMPPQAQQQQ    294
02QNg           VPDTGLPNSASVEQIQSKMQELSDLLEELRDSFDGYIS-NAFAGQIQLNFVMPPQAQQQQ    339
Taitung-4       VPDTGLPNSASIEQIQRKMQELNDVLEGLRDAFDGYIN-NAFVDQIQLNFVMPPQAQQQQ    345
IMRS_RE938      VPDTGLPNSASVEQIQRKMQELNDVLEGLRDAFDGYIN-NAFVDQIQLNFVMPPQAQQQQ    323
IMRS_RE1132     VPDTGLPNSASVEQIQRKMQELNDVLEGLRDAFDGYIN-NAFVDQIQLNFVMPPQAQQQQ    345
CMCOt1          VPDTGLPNSASVEQIQRKMQELNDVLEGLRDAFDGYIN-NAFVDQIQLNFVMPPQAQQQQ    345
IMRS_RE921      VPDTGLPNSASVEQIQRKMQELNDVLEGLRDAFDGYIN-NAFVDQIQLNFVMPPQAQQQQ    342
CMCOT7          VPDTSLPNSASVEQIQNKMQELNDVLEELRESFDGYLG-NAFADQIQLNFVMPPQAQQQQ    345
CMCOT11         VPDTSLPNSASVEQIQNKMQELNDVLEELRESFDGYLG-NAFADQIQLNFVMPPQAQQQQ    345
                ***:.*****::**** *::**.::** **::*:**:. ***.. ******** *** **
```

```
Jin/2012         GQGQQQQAQATVQEAVAAAAVRLLNGNDQIAQLYRDLVKLQRHAGIKKAMEKLAAQQEED         408
IMRS_RE215       GQGQQQQAQATAQEAVAAAAVRLLNGNNQ-------------------------------         374
UT150            GQGQQQQAQATAQEAVAAAAVRLLNGNNQIEQLYRDLVKLQRHAGIKKAMEKLAAQQEED         407
IMRS_RE814       GQGQQQQAQATAQEAVAAAAVRLLNGNNQI------------------------------         364
Taitung-7        GQGQQQQAQATAQEAVAAAAVRLLNGNDQIAQLYKDLVKLQRHAGIKKAMEKLAAQQEED         405
UT213            GQGQQQQAQATLQEAVAAAAVRLLNGNDQIAQLYKDLVKLQRHAGIRKAMEKLAAQQEED         408
IMRS_RE1044      GQGQQQQAQATLQEAVAAAAVRLLNGNDQ-------------------------------         362
Je-cheon         GQGQQQQAQATAQEAVAAAAVRLLNGNDQIEQLYKDLVKLQRHAGIKKAMEKLAAQQEED         405
CBNU-20          GQGQQQEAQATAQEAVAAAAVRLLNGNDQIAQLYKDLVKLQRHAGIKKAMEKLAAQQEED         405
T0224198         GQGQQQQAQATAQEAVAAAAVRLLNCNDQIAQLYKDLVKLQRHAGIKKAMEKLAAQQEED         408
UT176            GQGQQQQAQATAQEAVAAAAVRLLNGNDQIAQLYKDLVKLQRHAGIKKAMEKLAAQQEE-         404
Hualien-12       GQGQQQQAQATAQEAVAAAAVRLLNGNDQIAKLYKDLVKSQRHAGIKKAMEKLAAQQEE-         405
IMRS_RE626       GQGQQQQAQATAQEAVAAAAVRLLNGNDQ-------------------------------         374
IMRS_RE425       GQGQQQQAQATAQEAVAAAAVR--------------------------------------         365
IMRS_RE1079      GQGQQQQAQATAQEAVAAAAVRLLNGNDQ-------------------------------         373
IMRS_RE1163      GQGQQQQAQATAQEAVAAAAVRLLNGNDQ-------------------------------         364
IMRS_RE1028      GQGQQQQAQATAQEAVAAAAVRLLNGNDQ-------------------------------         375
IMRS_RE641       GQGQQQQAQATAQEAVAAAAVRLLNGNDQ-------------------------------         374
IMRS_RE933       GQGQQQQAQATAQEAVAAAAVRL-------------------------------------         367
LA-1             GQGQQQQAQATAQEAVAAAAVRLLNGNDHIAQLYKDLVKLQRHAGIKKAMEKLAAQQEE-         404
CMCOT2           GQGQQQQAQATAQEAVAAAAVRLLNGNDQIVQLYKDLVKLQRHAGIKKAMEKLAAQQEE-         404
05QNg            GQGQQQQAQATAQEAVAAAAVRLLNGNDQIVQLYKDLVKLQRHAGIKKAMEKLAAQQEE-         403
S0902151         GQGQQQQAQATAQEAVAAAAVRLLNGNDQIVQLYKDLVKLQRHAGIKKAMEKLAAQQEE-         403
01QNg            GQGQQQQAQATAQEAVAAAAVRLLNGNDQIVQLYKDLVKLQRHAGIKKAMEKLAAQQEE-         403
T1125175         GQGQQQQAQATAQEAVAAAAVRLLNGNDQIVQLYKDLVKLQRHAGIRKAMEKLAAQQEED         354
02QNg            GQGQQQQAQATAQEAVAAAAVRLLNGNDQIVQLYKDLVKLQRHAGIRKAMEKLAAQQEED         399
Taitung-4        VQRQQQQAQATAQEAVAATAVRLLNGNDQIVQLYKDLVKLQRHAGIKKAMEKLAAQQEED         405
IMRS_RE938       GQGQQQQAQATVQEAVAAAAVRLLNGKI--------------------------------         351
IMRS_RE1132      GQGQQQQAQATVQEAVAAAAVRLLNGNDQ-------------------------------         374
CMCOt1           GQGQQQQAQATAQEAVAAAAVRLLNGNDQIVQLYKDLVKLQRHAGIKKAMEKLAAQQEED         405
IMRS_RE921       GQGQQQQAQATVQEAVAAAAVRLLNGNDQ-------------------------------         371
CMCOT7           GQGQQQQGQATAQEAVAAAAVRLLNGNDQIAQLYKDLVKLQRHAGIKKAMEKLAAQQEED         405
CMCOT11          GQGQQQQGQATAQEAVAAAAVRLLNGNDQIAQLYKDLVKLQRHAGIKKAMEKLAAQQEED         405
                  * ***:.*** ******:***

Jin/2012         A------KNQGEGDCKQQQGASEKSKK-GKDKEAEFDLSMIVGQVKLYADLFATESFSIY         461
IMRS_RE215       ------------------------------------------------------------         374
UT150            A------KNQGEGDCKQQQGASEKSKEGGKGKEAEFDLSMIVGQVKLYADLMTTESFSIY         461
IMRS_RE814       ------------------------------------------------------------         364
Taitung-7        AKN----QGEGEGDCKQQQGASEKSKK-GKDKEAEFDLSMIVGQVKLYADLFTTESFSIY         460
UT213            AKN----QG--EGDCKQQQGASERSKE-GKGKEAEFDLSMIVGQVKLYADLFTTESFSVY         461
IMRS_RE1044      ------------------------------------------------------------         362
Je-cheon         AKN----QG--EGDCKQQQGASEESKK-GKDKEAEFDLNMIVGQVKLYADIVTTESFSIY         458
CBNU-20          AKN----QG--EGDCKQQQGASEKSKK-GKNEEAEFDLRMIVGQVKLYADIVTTESFSVY         458
T0224198         AKKEEDAKNQGEGDCKQQQGASEKSKEGGKGKEAEFDLSMIVGQVKLYADLFTTESFSIY         468
UT176            -----DAKNQGEGDCKQQQGASEKSKE-GKGKEAEFDLSMIVGQVKLYADLFTTESFSIY         458
Hualien-12       -----DAKNQGERDCKQQQGASEKSKK-GKDKEAEFDLSMIVGQVKLYADLFATESFSIY         459
IMRS_RE626       ------------------------------------------------------------         374
IMRS_RE425       ------------------------------------------------------------         365
IMRS_RE1079      ------------------------------------------------------------         373
IMRS_RE1163      ------------------------------------------------------------         364
IMRS_RE1028      ------------------------------------------------------------         375
IMRS_RE641       ------------------------------------------------------------         374
IMRS_RE933       ------------------------------------------------------------         367
LA-1             -----DAKNQGEGDCKQQQGTSEKSKEGS-KKEPEFDLSMIVGQVKLYADVMITESVSIY         458
CMCOT2           -----DAKNQGEGDCKQQQGASEKSKEGGKGKEAEFDLSMIVGQVKLYADVMITESFSVY         459
05QNg            -----DAKNQGEGDCKQQQGASEKSKEGGKGKEAEFDLSMIVGQVKLYADVMITESFSIY         458
S0902151         -----DAKNQGEGDCKQQQGASEKSKEGGKGKEAEFDLSMIVGQVKLYADVMITESFSIY         458
01QNg            -----DAKNQGEGDCKQQQGASEKSKEGGKGKEAEFDLSMIVGQVKLYADVMITESFSIY         458
T1125175         A------KNQGEGDCKQQQGASEKSKEGGKGKEAEFDLSMIVGQVKLYADVMITESFSIY         408
02QNg            S------KNQGEGSCKVDS------SKEGKSKETEFDLSMIVGQVKLYADLMTTESFSIY         447
Taitung-4        A------KNQGEGDCKQQQGASEKSKEGGKGKEAEFDLSMIVGQVKLYADLMTTESFSIY         459
IMRS_RE938       ------------------------------------------------------------         351
IMRS_RE1132      ------------------------------------------------------------         374
CMCOt1           A------KNQGEGDCKQQQGASEKSKEGGKGKEAEFDLSMIVGQVKLYADVMITESFSVY         459
IMRS_RE921       ------------------------------------------------------------         371
CMCOT7           A------KNQGEGDCKQQQGTSEKSKEGRKGKEAEFDLSMIVGQVKLYADLMTTESFSIY         459
CMCOT11          A------KNQGEGDCKQQQGTSEKSKEGSKGKEAEFDLSMIVGQVKLYADLMTTESFSIY         459
```

Jin/2012	AGLGAGLAYTYGKIDNKDIKGHTGMVASGALGVAINAAEGVYVDIEGSYMYSFSKIEEKY	521
IMRS_RE215	--	374
UT150	AGVGAGLAYTSGKIDGVDIKANTGMVASGALGVAINAAEGVYVDIEGGYMHSFSKIEEKY	521
IMRS_RE814	--	364
Taitung-7	AGLGAGLAYTSGKIDGVDIKANTGMVASGALGVAINAAEGVYVDIEGSYMHSFSKIEEKY	520
UT213	AGLGAGLAYTYGKIDNKDIKGHTGMVASGALGVAINAAEGVYVDIEGGYMHSFSKIEEKY	521
IMRS_RE1044	--	362
Je-cheon	AGVGAGVAYTYGKIDNKDIKGHTGMVASGALGVAINAAEGVCVDLEAGYMHSFSKVEDKY	518
CBNU-20	AGVGAGLAYTSGKIDGVDIKANTGMVASGALGVAINAADGVYVDIESSYMHSFSKVEDKY	518
T0224198	AGVGAGLAYTYGKIDNKDIKGHTGMVASGALGVAINAAEGVYVDIEGSYMYSFSKIEEKY	528
UT176	AGLGAGLAYTYGKIDNKDIKGHTGMVASGALGVAINAAEGVYVDIEGGYMHSFSKIEEKY	518
Hualien-12	AGLGAGLAYTYGKIDNKDIKGHTGMVASGALGVAINVAEGVCVDIEGSYMHSFSKIEEKY	519
IMRS_RE626	--	374
IMRS_RE425	--	365
IMRS_RE1079	--	373
IMRS_RE1163	--	364
IMRS_RE1028	--	375
IMRS_RE641	--	374
IMRS_RE933	--	367
LA-1	AGVGAGLAYTSGKIDDKDT---	477
CMCOT2	AGVGAGLAYTYGKIDNKDIKGHTGMVASRALGVAINAAEGVYVDIESGYMYSFSKIEEKY	519
05QNg	AGVGAGLAYTYGKIDNKDIKGHTGMVASGALGVAINAAEGVYVDIEGSYMYSFSKIEEKY	518
S0902151	AGVGAGLAYTYGKIDNKDIKGHTGMVASGALGVAINAAEGVYVDIEGSYMYSFSKIEEKY	518
01QNg	AGVGAGLAYTYGKIDNKDIKGHTGMVASGALGVAINAAEGVYVDIEGSYMYSFSKIEEKY	518
T1125175	AGVGAGLAYTYGKIDNKDIKGHTGMVASGALGVAINAAEGVYVDIEGSYMYSFSKIEEKY	468
02QNg	AGLGAGLAYTYGKIDDKDI-GHTGMVASGALGVAINAAEGVYVDIEGGYMYSFSKIEEKY	506
Taitung-4	AGVGAGLAYTYGKIDNKDIKGHTGMVASGALGVAINVAEGVCVDIEGSYMHSFSKIEEKY	519
IMRS_RE938	--	351
IMRS_RE1132	--	374
CMCOt1	AGVGAGLAYTYGKIDNKDIKGHTGMVASGALGVAINAAEGVYVDIEGGYMYSFSKIEEKY	519
IMRS_RE921	--	371
CMCOT7	AGVGAGVAYTYGKIDNKDIKGHTGMVASGALGVAINAAEGVYVDIEGGYMHSFSKIEEKY	519
CMCOT11	AGVGAGVAYTYGKIDNKDIKGHTGMVASGALGVAINAAEGVYVDIEGGYMHSFSKIEEKY	519

Jin/2012	SINPLMASFGVRYNF	536
IMRS_RE215	---------------	374
UT150	SVNALMASAGVRYNF	536
IMRS_RE814	---------------	364
Taitung-7	SVNALMANIGVRYNF	535
UT213	SVNAIMASAGVRYNF	536
IMRS_RE1044	---------------	362
Je-cheon	QVNALIASASVRYNF	533
CBNU-20	S--------------	519
T0224198	SINPLMASAGVR---	540
UT176	SVNAVMASAGVRYNF	533
Hualien-12	SVNALMANVGVRYNF	534
IMRS_RE626	---------------	374
IMRS_RE425	---------------	365
IMRS_RE1079	---------------	373
IMRS_RE1163	---------------	364
IMRS_RE1028	---------------	375
IMRS_RE641	---------------	374
IMRS_RE933	---------------	367
LA-1	---------------	477
CMCOT2	PINPLMASARV----	530
05QNg	SINPLMASAG-----	528
S0902151	SINPLMASA------	527
01QNg	SINPLMASA------	527
T1125175	SINPLMASA------	477
02QNg	SINPLMASA------	515
Taitung-4	SVNALMANVGVRYNF	534
IMRS_RE938	---------------	351
IMRS_RE1132	---------------	374
CMCOt1	SINPLMA--------	526
IMRS_RE921	---------------	371
CMCOT7	SVNALMASAGVRYNF	534
CMCOT11	SVNALMASAGVRYNF	534

B.

```
#
#
#  Percent Identity  Matrix - created by Clustal2.1
#
#
```

1: Jin/2012	100.00	96.52	94.58	96.43	88.78	89.29	89.30	87.21	87.25	91.05	90.46	91.05	91.28	89.66	90.16	89.92	90.22	90.19	90.00	89.94	89.81	91.51	91.88	91.68	84.29	83.10	77.32	70.89	73.24	78.89	73.30	84.50	85.44
2: IMRS_RE215	96.52	100.00	100.00	100.00	88.19	89.56	90.14	89.29	88.46	89.89	89.32	89.32	91.51	91.04	91.23	91.01	91.23	91.23	91.36	91.48	90.38	91.21	91.76	91.48	80.44	80.83	71.93	71.39	73.30	73.57	73.50	82.02	83.38
3: UT150	94.58	100.00	100.00	100.00	89.52	89.87	90.14	88.15	88.61	90.48	90.65	88.74	91.53	91.04	91.23	91.01	91.28	91.26	91.36	90.79	90.00	91.33	91.70	91.51	83.86	82.12	78.26	71.39	73.44	78.89	73.50	85.63	86.58
4: IMRS_RE814	96.43	100.00	100.00	100.00	87.85	89.27	90.08	88.98	88.14	89.61	89.01	89.01	91.24	90.78	90.96	90.96	90.96	90.96	91.09	91.24	90.11	91.24	91.53	91.24	80.50	80.29	71.15	71.39	72.47	72.83	72.75	81.51	82.91
5: Taitung-7	88.78	88.19	89.52	87.85	100.00	93.23	94.75	91.74	93.26	87.03	89.25	89.08	88.74	89.01	89.25	89.26	89.57	89.54	89.34	88.61	87.67	89.90	90.08	90.08	81.41	80.83	78.67	72.59	74.86	78.53	74.93	83.43	84.19
6: UT213	89.29	89.56	89.87	89.27	93.23	100.00	99.17	91.54	90.73	88.09	90.91	88.64	89.52	89.26	89.49	89.50	89.81	89.78	89.59	88.75	90.08	91.40	91.57	91.57	82.15	81.51	79.89	73.98	76.44	80.74	76.52	84.67	85.44
7: IMRS_RE1044	89.30	90.14	90.14	90.08	94.75	99.17	100.00	93.92	92.82	87.29	88.95	88.95	90.06	89.58	89.78	90.06	90.06	90.06	89.89	90.88	91.16	92.82	92.82	92.82	78.98	79.60	74.86	74.56	76.27	76.27	76.55	81.36	82.49
8: Je-cheon	87.21	89.29	88.15	88.98	91.74	91.54	93.92	100.00	93.26	86.98	88.26	87.71	89.81	89.29	89.52	89.53	89.84	89.81	89.62	88.35	88.38	89.67	90.04	90.04	80.26	79.37	77.44	72.30	74.59	77.86	74.66	83.17	83.94
9: CBNU-20	87.25	88.46	88.61	88.14	93.26	90.73	92.82	93.26	100.00	85.93	86.58	86.02	87.94	87.64	87.90	87.88	88.24	88.20	87.98	87.50	87.38	88.52	88.72	88.72	79.26	78.23	76.03	71.14	73.50	77.01	73.55	81.73	82.51
10: T0224198	91.05	89.89	90.48	89.61	87.03	88.09	87.29	86.98	85.93	100.00	95.09	92.28	95.72	88.77	88.74	88.74	89.07	89.04	89.10	88.89	89.43	91.86	92.03	92.03	84.22	80.87	80.31	73.76	75.96	82.63	76.03	83.56	84.70
11: UT176	90.46	89.32	90.65	89.01	89.25	90.91	88.95	88.26	86.58	95.09	100.00	94.56	96.25	92.03	92.20	92.29	92.51	92.49	92.35	90.11	89.20	92.03	92.40	92.21	83.87	82.50	80.27	73.39	75.89	80.93	75.69	85.82	86.97
12: Hualien-12	91.05	89.32	88.74	89.01	89.08	88.64	88.95	87.71	86.02	92.28	94.56	100.00	95.72	89.86	90.08	90.11	90.40	90.37	90.19	88.45	87.52	90.13	90.49	90.30	82.62	81.55	79.92	72.30	74.59	78.83	74.66	83.56	84.70
13: IMRS_RE626	91.28	91.51	91.53	91.24	88.74	89.52	90.06	89.81	87.94	95.72	96.25	95.72	100.00	90.96	91.15	91.21	91.44	91.44	91.28	90.08	89.28	92.74	93.28	93.01	79.05	79.94	73.15	72.30	74.52	74.79	74.66	81.64	83.29
14: IMRS_RE425	89.66	91.04	91.04	90.78	89.01	89.26	89.58	89.29	87.64	88.77	92.03	89.86	90.96	100.00	99.45	99.72	99.73	99.73	99.73	94.51	92.86	95.32	95.87	95.59	79.87	80.29	72.75	73.00	74.16	74.44	74.44	82.58	84.27
15: IMRS_RE1079	90.16	91.23	91.23	90.96	89.25	89.49	89.78	89.52	87.90	88.74	92.20	90.08	91.15	99.45	100.00	99.73	99.73	99.73	99.73	94.35	93.01	95.42	95.96	95.69	80.32	80.73	73.35	72.89	74.73	75.00	74.93	82.97	84.62
16: IMRS_RE1163	89.92	91.01	91.01	90.96	89.26	89.50	90.06	89.53	87.88	88.74	92.29	90.11	91.21	99.72	99.73	100.00	100.00	100.00	100.00	94.49	93.11	95.86	96.13	95.86	80.63	80.52	72.96	73.18	74.37	74.65	74.65	82.82	84.51
17: IMRS_RE1028	90.22	91.23	91.28	90.96	89.57	89.81	90.06	89.84	88.24	89.07	92.51	90.40	91.44	99.73	99.73	100.00	100.00	100.00	100.00	94.65	93.32	95.71	96.25	95.98	80.63	81.11	73.77	73.18	75.14	75.41	75.21	83.33	84.97
18: IMRS_RE641	90.19	91.23	91.26	90.96	89.54	89.78	90.06	89.81	88.20	89.04	92.49	90.37	91.44	99.73	99.73	100.00	100.00	100.00	100.00	94.64	93.30	95.70	96.24	95.97	80.63	81.06	73.70	73.18	75.07	75.34	75.21	83.29	84.93
19: IMRS_RE933	90.00	91.36	91.36	91.09	89.34	89.59	89.89	89.62	87.98	89.10	92.35	90.19	91.28	99.73	99.73	100.00	100.00	100.00	100.00	94.81	93.17	95.62	96.16	95.89	80.26	80.68	73.18	73.37	74.58	74.86	74.79	82.96	84.64
20: LA-1	89.94	91.48	90.79	91.24	88.61	88.75	90.88	88.35	87.50	88.89	90.11	88.45	90.08	94.51	94.35	94.49	94.65	94.64	94.81	100.00	93.92	94.75	95.17	94.96	84.13	82.20	78.16	74.64	76.50	79.66	76.58	85.22	86.72
21: CMCOT2	89.81	90.38	90.00	90.11	87.67	90.08	91.16	88.38	87.38	89.43	89.20	87.52	89.28	92.86	93.01	93.11	93.32	93.30	93.17	93.92	100.00	94.89	95.45	95.26	86.08	84.16	81.35	76.97	78.96	84.69	79.06	86.54	87.69
22: 05QNg	91.51	91.21	91.33	91.24	89.90	91.40	92.82	89.67	88.52	91.86	92.03	90.13	92.74	95.32	95.42	95.86	95.71	95.70	95.62	94.75	94.89	100.00	99.62	99.81	87.12	83.93	80.89	74.85	76.71	83.30	76.80	86.49	87.45
23: S0902151	91.88	91.76	91.70	91.53	90.08	91.57	92.82	90.04	88.72	92.03	92.40	90.49	93.28	95.87	95.96	96.13	96.25	96.24	96.16	95.17	95.45	99.62	100.00	99.81	87.34	84.33	81.04	74.85	76.99	83.50	77.07	86.85	87.81
24: 01QNg	91.68	91.48	91.51	91.24	90.08	91.57	92.82	90.04	88.72	92.03	92.21	90.30	93.01	95.59	95.69	95.86	95.98	95.97	95.89	94.96	95.26	99.81	99.81	100.00	87.12	84.13	81.04	74.85	76.99	83.50	77.07	86.65	87.62
25: T1125175	84.29	80.44	83.86	80.50	81.41	82.15	78.98	80.26	79.26	84.22	83.87	82.62	79.05	79.87	80.32	80.63	80.63	80.63	80.26	84.13	86.08	87.12	87.34	87.12	100.00	94.47	83.61	79.44	80.43	86.29	80.75	84.66	84.45
26: 02QNg	83.10	80.83	82.12	80.29	80.83	81.51	79.60	79.37	78.23	80.87	82.50	81.55	79.94	80.29	80.73	80.52	81.11	81.06	80.68	82.20	84.16	83.93	84.33	84.13	94.47	100.00	81.32	78.78	80.93	83.40	81.04	83.66	83.46
27: Taitung-4	77.32	71.93	78.26	71.15	78.67	79.89	74.86	77.44	76.03	80.31	80.27	79.92	73.15	72.75	73.35	72.96	73.77	73.70	73.18	78.16	81.35	80.89	81.04	81.04	83.61	81.32	100.00	95.16	95.99	96.39	97.30	88.01	85.58
28: IMRS_RE938	70.89	71.39	71.39	71.39	72.59	73.98	74.56	72.30	71.14	73.76	73.39	72.30	72.30	73.00	72.89	73.18	73.18	73.18	73.37	74.64	76.97	74.85	74.85	74.85	79.44	78.78	95.16	100.00	96.58	97.44	98.01	85.19	81.48
29: IMRS_RE1132	73.24	73.30	73.44	72.47	74.86	76.44	76.27	74.59	73.50	75.96	75.89	74.59	74.52	74.16	74.73	74.37	75.14	75.07	74.58	76.50	78.96	76.71	76.99	76.99	80.43	80.93	95.99	96.58	100.00	98.13	98.65	86.36	82.89
30: CMCOt1	78.89	73.57	78.89	72.83	78.53	80.74	76.27	77.86	77.01	82.63	80.93	78.83	74.79	74.44	75.00	74.65	75.41	75.34	74.86	79.66	84.69	83.30	83.50	83.50	86.29	83.40	96.39	97.44	98.13	100.00	99.46	89.16	86.50
31: IMRS_RE921	73.30	73.50	73.50	72.75	74.93	76.52	76.55	74.66	73.55	76.03	75.69	74.66	74.66	74.44	74.93	74.65	75.21	75.21	74.79	76.58	79.06	76.80	77.07	77.07	80.75	81.04	97.30	98.01	98.65	99.46	100.00	87.33	83.56
32: CMCOT7	84.50	82.02	85.63	81.51	83.43	84.67	81.36	83.17	81.73	83.56	85.82	83.56	81.64	82.58	82.97	82.82	83.33	83.29	82.96	85.22	86.54	86.49	86.85	86.65	84.66	83.66	88.01	85.19	86.36	89.16	87.33	100.00	96.63
33: CMCOT11	85.44	83.38	86.58	82.91	84.19	85.44	82.49	83.94	82.51	84.70	86.97	84.70	83.29	84.27	84.62	84.51	84.97	84.93	84.64	86.72	87.69	87.45	87.81	87.62	84.45	83.46	85.58	81.48	82.89	86.50	83.56	96.63	100.00

CLUSTAL O(1.2.4) multiple sequence alignment

Kato

```
IMRS_RE1160      CTAGAACTGATACAAACTCTCCTATGCCTCCGCGGTATAAACTTACGCCACCTCAGCCTA   232
RE1160           ------------------------------------------------TTCCTCAGCCTA   12
IMRS_RE1161      CTAGAACTGATACAAACTCTCCTATGCCTCCGCGGTATAAACTTACGCCACCTCAGCCTA   414
RE1161           ------------------------------------------------TTCCTCAGCCTA   12
IMRS_RE1071      CTAGAATTAATACAGGTTCTCCTATGCCTCCGCGGTATAAACTTACGCCACCTCAGCCTA   400
RE1071           ------------------------------------------------TTCCTCAGCCTA   12
IMRS_RE1072      CTAGAATTAATACAGGTTCTCCTATGCCTCCGCGGTATAAACTTACGCCACCTCAGCCTA   246
RE1072           ------------------------------------------------TTCCTCAGCCTA   12
                                                                 ************

IMRS_RE1160      CTATAATGCCTATAAGTATAGCTGATCGTGACCTTGGGGTTGATATTCCTAACGTACCTC   292
RE1160           CTATAATGCCTATAAGTATAGCTGATCGTGACCTTGGGGTTGATATTCCTAACGTACCTC   72
IMRS_RE1161      CTATAATGCCTATAAGTATAGCTGATCGTGACCTTGGGGTTGATATTCCTAACGTACCTC   474
RE1161           CTATAATGCCTATAAGTATAGCTGATCGTGACCTTGGGGTTGATATTCCTAACGTACCTC   72
IMRS_RE1071      CTATAATGCCTATAAGTATAGCTGATCGTGACTTTGGGGTTGATGTTGTTAATGTGCCTC   460
RE1071           CTATAATGCCTATAAGTATAGCTGATCGTGACTTTGGGGTTGATGTTGTTAATGTGCCTC   72
IMRS_RE1072      CTATAATGCCTATAAGTATAGCTGATCGTGACTTTGGGGTTGATGTTGTTAATGTGCCTC   306
RE1072           CTATAATGCCTATAAGTATAGCTGATCGTGACTTTGGGGTTGATGTTGTTAATGTGCCTC   72
                 ******************************** *********** **  *** ** ****

IMRS_RE1160      AAGGAGGAGCTAATCACCTGGGTAATAACCTTGGTGCTAATGATATTCGGCGTGCTGCTG   352
RE1160           AAGGAGGAGCTAATCACCTGGGTAATAACCTTGGTGCTAATGATATTCGGCGTGCTGCTG   132
IMRS_RE1161      AAGGAGGAGCTAATCACCTGGGTAATAACCTTGGTGCTAATGATATTCGGCGTGCTGCTG   534
RE1161           AAGGAGGAGCTAATCACCTGGGTAATAACCTTGGTGCTAATGATATTCGGCGTGCTGCTG   132
IMRS_RE1071      AAAATCAAGTGCAGGC---------AAATCAGGTGAATGATCCTCTTGTTCGTGGAGGAC   511
RE1071           AAAATCAAGTGCAGGC---------AAATCAGGTGAATGATCCTCTTGTTCGTGGAGGAC   123
IMRS_RE1072      AAAATCAAGTGCAGGC---------AAATCAGGTGAATGATCCTCTTGTTCGTGGAGGAC   357
RE1072           AAAATCAAGTGCAGGC---------AAATCAGGTGAATGATCCTCTTGTTCGTGGAGGAC   123
                 **     **   *             ** *  *    * **  * **   ****  *

IMRS_RE1160      ATAGGATCGCTTGGTTGAAGAATTATGCTGGTATTGACTATATGGTTCCAGATCCTCAGA   412
RE1160           ATAGGATCGCTTGGTTGAAGAATTATGCTGGTATTGACTATATGGTTCCAGATCCTCAGA   192
IMRS_RE1161      ATAGGATCGCTTGGTTGAAGAATTATGCTGGTATTGACTATATGGTTCCAGATCCTCAGA   594
RE1161           ATAGGATCGCTTGGTTGAAGAATTATGCTGGTATTGACTATATGGTTCCAGATCCTCAGA   192
IMRS_RE1071      GTAGAATTGCTTGGTTAAAAGAGTATGCTGGTATTGACTATATGGTGAAGGATCCTGATA   571
RE1071           GTAGAATTGCTTGGTTAAAAGAGTATGCTGGTATTGACTATATGGTGAAGGATCCTGATA   183
IMRS_RE1072      GTAGAATTGCTTGGTTAAAAGAGTATGCTGGTATTGACTATATGGTGAAGGATCCTGATA   417
RE1072           GTAGAATTGCTTGGTTAAAAGAGTATGCTGGTATTGACTATATGGTGAAGGATCCTGATA   183
                  *** ** ******** **  * ***********************    ****** * *

IMRS_RE1160      ATCCTCAGGCTAGAGTTGTAAATCCAGTGCTATTAGATATTCCTCAAGGTCCACCTAATG   472
RE1160           ATCCTCAGGCTAGAGTTGTAAATCCAGTGCTATTAGATATTCCTCAAGGTCCACCTAATG   252
IMRS_RE1161      ATCCTCAGGCTAGAGTTGTAAATCCAGTGCTATTAGATATTCCTCAAGGTCCACCTAATG   654
RE1161           ATCCTCAGGCTAGAGTTGTAAATCCAGTGCTATTAGATATTCCTCAAGGTCCACCTAATG   252
IMRS_RE1071      ATCCTGGGCATATGATGATAAATCCGGTGTTGTTAGATATTCCTCAAGGTAACCCTGCTA   631
RE1071           ATCCTGGGCATATGATGATAAATCCGGTGTTGTTAGATATTCCTCAAGGTAACCCTGCTA   243
IMRS_RE1072      ATCCTGGGCATATGATGATAAATCCGGTGTTGTTAGATATTCCTCAAGGTAACCCTGCTA   477
RE1072           ATCCTGGGCATATGATGATAAATCCGGTGTTGTTAGATATTCCTCAAGGTAACCCTGCTA   243
                 *****  *  **   *  ******* *** * ******************   ***  *

IMRS_RE1160      CAAATCCTAGACAAGCTGTGCAACCTTGTAGAATACTTGACCATGAACACTGGAGACATC   532
RE1160           CAAATCCTAGACAAGCTGTGCAACCTTGTAGAATACTTGACCATGAACACTGGAGACATC   312
IMRS_RE1161      CAAATCCTAGACAAGCTGTGCAACCTTGTAGAATACTTGACCATGAACACTGGAGACATC   714
RE1161           CAAATCCTAGACAAGCTGTGCAACCTTGTAGAATACTTGACCATGAACACTGGAGACATC   312
```

```
IMRS_RE1071      ACAATCCTAGAGCGGCTTTGCAGCCTTGTAGTATACTTGACCATGCACAATGGAGGGATA   691
RE1071           ACAATCCTAGAGCGGCTTTGCAGCCTTGTAGTATACTTGACCATGCACAATGGAGGGATA   303
IMRS_RE1072      ACAATCCTAGAGCGGCTTTGCAGCCTTGTAGTATACTTGACCATGCACAATGGAGGGATA   537
RE1072           ACAATCCTAGAGCGGCTTTGCAGCCTTGTAGTATACTTGACCATGCACAATGGAGGGATA   303
                   *********   *** **** ******** ************* *** *****  **

IMRS_RE1160      TTGTAGTTGGTGTTACTGCAATGTCAAATGCTAATAAACCTAGCGTTTCTCCTGTCAAAG   592
RE1160           TTGTAGTTGGTGTTACTGCAATGTCAAATGCTAATAAACCTAGCGTTTCTCCTGTCAAAG   372
IMRS_RE1161      TTGTAGTTGGTGTTACTGCAATGTCAAATGCTAATAAACCTAGCGTTTCTCCTGTCAAAG   774
RE1161           TTGTAGTTGGTGTTACTGCAATGTCAAATGCTAATAAACCTAGCGTTTCTCCTGTCAAAG   372
IMRS_RE1071      TGGTAGTTGGTATTACTGCAATGTCAAATGCTAATAAACCTAGCGCTTCTCCTATCAAAG   751
RE1071           TGGTAGTTGGTATTACTGCAATGTCAAATGCTAATAAACCTAGCGCTTCTCCTATCAAAG   363
IMRS_RE1072      TGGTAGTTGGTATTACTGCAATGTCAAATGCTAATAAACCTAGCGCTTCTCCTATCAAAG   597
RE1072           TGGTAGTTGGTATTACTGCAATGTCAAATGCTAATAAACCTAGCGCTTCTCCTATCAAAG   363
                 * ********* ********************************* ******* ******

IMRS_RE1160      TATTAAGTGACAAAATTGTCCAGATATATCGTGATATAAAGCCGTTTGCTAGAGTAGCTG   652
RE1160           TATTAAGTGACAAAATTGTCCAGATATATCGTGATATAAAGCCGTTTGCTAGAGTAGCTG   432
IMRS_RE1161      TATTAAGTGACAAAATTGTCCAGATATATCGTGATATAAAGCCGTTTGCTAGAGTAGCTG   834
RE1161           TATTAAGTGACAAAATTGTCCAGATATATCGTGATATAAAGCCGTTTGCTAGAGTAGCTG   442
IMRS_RE1071      TATTAAGTGATAAAATTAGTCAGATATATGATGATATAAGGCCATTTGCTGAGATAGCTG   811
RE1071           TATTAAGTGATAAAATTAGTCAGATATATGATGATATAAGGCCATTTGCTGAGATAGCTG   423
IMRS_RE1072      TATTAAGTGATAAAATTAGTCAGATATATGATGATATAAGGCCATTTGCTGAGATAGCTG   657
RE1072           TATTAAGTGATAAAATTAGTCAGATATATGATGATATAAGGCCATTTGCTGAGATAGCTG   423
                 ********** ******   *********  ******** *** ******    ******

IMRS_RE1160      GTATTGAAGTTCCTAGTGATCCTTTGCCTAATAGTGCATCTGTTGAGCAGATACAGAATA   712
RE1160           GTATGAAGTCCTA-----------------------------------------------   445
IMRS_RE1161      GTATTGAAGTTCCTAGTGATCCTTTGCCTAATAGTGCATCTGTTGAGCAGATACAGAATA   894
RE1161           GTATGAAGT---------------------------------------------------   441
IMRS_RE1071      GTATTAACATTCCTGATACTGTTTTGCCTAATAGTGCATCTGTTGAGCAGATACAGAGTA   871
RE1071           GTATTAACATTCCTGATACTGTTTTGCCTAATAGTGCATCTGTCGGGATCCCTAGA----   479
IMRS_RE1072      GTATTAACATTCCTGATACTGTTTTGCCTAATAGTGCATCTGTTGAGCAGATACAGAGTA   717
RE1072           GTATTAACATTCCTGATACTGTTTTGCCTAATAGTGCATCTGTCGGGATCCCTAGA----   479
                 ****  *
```

Karp

```
IMRS_RE814       CAGCCTACTATAATGCCTATAAGTATAGCTGATCGTGACTTTGGGATTGATATTCGTAAC   456
RE814            CAGCCTACTATAATGCCTATAAGTATAGCTGATCGTGACTTTGGGATTGATATTCGTAAC   64
IMRS_RE1163      CAGCCTACTATAATGCCTATAAGTATAGCTGATCGTGACTTTGGGATTGATATTTGTAAC   438
RE1163           CAGCCTACTATAATGCCTATAAGTATAGCTGATCGTGACTTTGGGATTGATATTTGTAAC   64
IMRS_RE1044      CAGCCTACTATAATGCCTATAAGTATAGCTGATCGTGACTTTGGGATTGATATTCCTAAC   438
RE1044           CAGCCTACTATAATGCCTATAAGTATAGCTGATCGTGACTTTGGGATTGATATTCCTAAC   64
IMRS_RE333       CAACCTACTATAATGCCTATAAGTATAGCAGATCGTGATTTCGGTGTTGATGTTACTAAT   456
RE333            ---------------------------------------TTCGGTGTTGATGTTACTAAT   21
                                                        ** **  ***** **  ***

IMRS_RE814       ATACCTCAGGCGCAAGCTGGG------------AATCTTAATAATGAGCAGCGTGCTGCA   504
RE814            ATACCTCAGGCGCAAGCTGGG------------AATCTTAATAATGAGCAGCGTGCTGCA   112
IMRS_RE1163      ATACCTCAGCAGCAAGCGCAAGCTGCGAATCCTGCGCTTAATGATGAACAGCGTGCTGCA   498
RE1163           ATACCTCAGCAGCAAGCGCAAGCTGCGAATCCTGCGCTTAATGATGAACAGCGTGCTGCA   124
IMRS_RE1044      ATACCTCAGCAGCAAGCACAAGCGGCGCAGCCTCAGCTTAATGATGAGCAACGTGCTGCA   498
RE1044           ATACCTCAGCAGCAAGCACAAGCGGCGCAGCCTCAGCTTAATGATGAGCAACGTGCTGCA   124
IMRS_RE333       ATACCTCAAGCTCAAGTACAACCGCCTCAGCAAGAAAATGATCCTCTTGTTCGTGGAGTA   516
RE333            ATACCTCAAGCTCAAGTACAACCGCCTCAGCCTGCAAATGATCCTCTTGTTCGTGGAGTA   81
                 ********    ****                      * **  *      ****  * *
```

```
IMRS_RE814       GCTAGGATCGCTTGGTTAAAGAATTGTGCTGGTATTGACTATAGGGTAAAAGATCCTAAT   564
RE814            GCTAGGATCGCTTGGTTAAAGAATTGTGCTGGTATTGACTATAGGGTAAAAGATCCTAAT   172
IMRS_RE1163      GCTAGGATCGCTTGGTTAAAGAATTGTGCTGGTATTGACTATAGGGTAAAAGATCCTAAT   558
RE1163           GCTAGGATCGCTTGGTTAAAGAATTGTGCTGGTATTGACTATAGGGTAAAAGATCCTAAT   184
IMRS_RE1044      GCTAGGATCGCTTGGTTAAAGAATTGTGCTGGTATTGACTATAGGGTAAAAAACCCTAAT   558
RE1044           GCTAGGATCGCTTGGTTAAAGAATTGTGCTGGTATTGACTATAGGGTAAAAAACCCTAAT   184
IMRS_RE333       CGTAGGATTGCTTGGGTAAAAGAGTATGCTGGTATTGACTATATGGGGAACGATCCTAAT   576
RE333            CGTAGGATTGCTTGGTTAAAAAAGTATGCTGGTATTGACTATATGGTGAAGGATCCTAAT   141
                   ****** ****** ****  * * ***************** **  **  * ******

IMRS_RE814       AATCCTAATGGGCCTATGGTTATAAATCCGATATTGTTAAATATTCCACAGGGTAACCCT   624
RE814            AATCCTAATGGGCCTATGGTTATAAATCCGATATTGTTAAATATTCCACAGGGTAACCCT   232
IMRS_RE1163      AATCCTAATGGGCCTATGGTTATAAATCCGATATTGTTAAATATTCCACAGGGTAACCCT   618
RE1163           AATCCTAATGGGCCTATGGTTATAAATCCGATATTGTTAAATATTCCACAGGGTAACCCT   244
IMRS_RE1044      GATCCTAATGGGCCTATGGTTATAAATCCGATATTGTTAAATATTCCACAGGGTAACCCT   618
RE1044           GATCCTAATGGGCCTATGGTTATAAATCCGATATTGTTAAATATTCCACAGGGTAACCCT   244
IMRS_RE333       AATCCTGGGCGTATGA---TGGTAAATCCAGTGTTGTTAAATATACCTCAAGGCCCACCT   633
RE333            AATCCTGGGCGTATGA---TGGTAAATCCTGTGATGTTAAATATACCTCCAGGCCCACCT   198
                  *****    *    *   *  *******  *  ********** ** *  **    ***

IMRS_RE814       ATTCCTGTTGGAAAT------CAGCGAGCACAGCAGCCTGCAGGGTTTGCGATACATGAC   678
RE814            ATTCCTGTTGGAAAT------CAGCGAGCACAGCAGCCTGCAGGGTTTGCGATACATGAC   286
IMRS_RE1163      AATCCTGCTGGGAATCCACCGCAGCGAGCACAGCAGCCTGCAAATTTTGCGATACATAAC   678
RE1163           AATCCTGCTGGGAATCCACCGCAGCGAGCACAGCAGCCTGCAAATTTTGCGATACATAAC   304
IMRS_RE1044      AATCCTGTTGGAAATCCACCGCAGCGAGCAAATCCGCCTGCAGGTTTTGCGATACATAAC   678
RE1044           AATCCTGTTGGAAATCCACCGCAGCGAGCAAATCCGCCTGCAGGTTTTGCGATACATAAC   304
IMRS_RE333       GCTCAAAAT---------------CCTACAGCGGCTATGCAACCTTGTAATATACGTGAT   678
RE333            GCTCAAAAT---------------CCTAGAGCGGCTATGCAACCTTGTAATATACTTGAT   243
                   **    *               *    *           *   * *   **** * *

IMRS_RE814       CATGAGCAATGGAGGTATTTGGTAACTGGTCTTGCTGCATTATCAAATGCTAATAAACCT   738
RE814            CATGAGCAATGGAGGTATTTGGTAACTGGTCTTGCTGCATTATCAAATGCTAATAAACCT   346
IMRS_RE1163      CATGATCAATGGAGGCATTTGGTAGTTGGGCTTGCTGCATTATCAAATGCTAATAAGCCT   738
RE1163           CATGATCAATGGAGGCATTTGGTAGTTGGGCTTGCTGCATTATCAAATGCTAATAAGCCT   364
IMRS_RE1044      CATGAGCAATGGAGGCATTTGGTAGTTGGGCTTGCTGCATTATCAAATGCTAATAAACCT   738
RE1044           CATGAGCAATGGAGGCATTTGGTAGTTGGGCTTGCTGCATTATCAAATGCTAATAAACCT   364
IMRS_RE333       CATGATCACTGGAGACATTTGGTAGTTGGCGTCACTGCATTATCAAATGCTAATAAACCT   738
RE333            CATGATCACTGGAGACATTTGGTACTTGGTGTCCCTGCATTATCAAATGCTAATAAACCT   303
                 ***** ** *****  ********  ***  *  ********************** ***

IMRS_RE814       AGCGCTTCTCCTGTCAAAGTATTAAGTGATAAAATTACTCAGATATATAGTGATATAAAG   798
RE814            AGCGCTTCTCCTGTCAAAGTATTAAGTGATAAAATTACTCAGATATATAGTGATATAAAG   406
IMRS_RE1163      AGCGCTTCTCCTGTCAAAGTATTAAGTGATAAAATTACTCAGATATATAGTGATATAAAG   798
RE1163           AGCGCTTCTCCTGTCAAAGTATTAAGTGATAAAATTACTCAGATATATAGTGATATAAAG   424
IMRS_RE1044      AGCGCTTCTCCTGTCAAAGTATTAAGTGATAAAATTTCTCAGATATATAGTGATATAAAG   798
RE1044           AGCGCTTCTCCTGTCAAAGTATTAAGTGATAAAATTTCTCAGATATATAGTGATATAAAG   424
IMRS_RE333       AGCGCTTCTCCTGTCAAAATATTAAGTGAAAAAATTACTCAGATATATAGTGATATAAGG   798
RE333            ACCGCTTCTCCTGTCCAAATATTAAGTGAAAAAATTACTCAAATATATAGTGATATAAGG   363
                 * ************* ** ********** ****** **** **************** *

IMRS_RE814       CTATTTGCTGATATAGCTGGTATTGATGTTCCTGATGCTGGTTTGCCTAATAGTGCAACT   858
RE814            CTATTTGCTGATATAGCTGGTATTGATGTTCCTGATGCTGGTTTGCCTAATAGTGCATCT   466
IMRS_RE1163      CCATTTGCTGATATAGCTGGTATTGATGTTCCTGATACTAGTTTGCCTAATAGTGCATCT   858
RE1163           CCATTTGCTGATATAGCTGGTATTGATGTTCCTGATACTAGTTTGCCTAATAGTGCATCT   484
IMRS_RE1044      CCATTTGCTGATATAGCTGGTATTGATGTTCCTGATACTGGTTTGCCTAATAGTGCATCT   858
RE1044           CCATTTGCTGATATAGCTGGTATTGATGTTCCTGATACTGGTTTGCCTAATAGTGCATCT   484
IMRS_RE333       CCATCTGCTGATATAGCTGGTATTAATGTTCCTGATACTGGTTTGCCTAATAGTGCGACT   858
RE333            ACATTTGCTGATATAGCTGGTATTAATGTTCCTGATACTGGTTTGCCTAATAATGCTTCT   423
                   ** ******************* *********** ** ************ ***  **
```

```
IMRS_RE814      GTCGAACAGATACAGAATAAAATACAAGAATTAAACGATGTATTGGAAGAGCTCAGAGAA   918
RE814           GTCG--------------------------------------------------------   470
IMRS_RE1163     GTTGAACAGATACAGAATAAAATGCAAGAATTAAACGATCTATTGGAAGAGCTCAGAGAA   918
RE1163          GT----------------------------------------------------------   486
IMRS_RE1044     GTCGAACAGATACAGAGTAAAATGCAAGAATTAAACGATGTATTGGAAGAACTCAGAGAT   918
RE1044          GTCG--------------------------------------------------------   488
IMRS_RE333      GTCGAACAGATACAGAGTAAAATGCAA---------------------------------   885
RE333           GTC---------------------------------------------------------   426
                **
```

TA763

```
IMRS_RE1132     GATGCAAATCAGGCAAAACATCTTAATCCACCTCCTAAACTTACACCGCCTCAACCTGTT   420
RE1132          ------------------------------------------------CCTCAACCTGTT   12
IMRS_RE451      GATGCAAATCAGGCAAAACAACTTAAGCGACCTCCTATGATCAAGCCTCCTCAGCCTACT   420
RE451           -----------------------------------------------TCCTCAGCCTACT   13
IMRS_RE773      -------------AGGCTCCTATACGTAAGCGGCCTAAACTTACACCACCTCAGCCTACT   399
RE773           -----------------------------------------------TCCTCAGCCTACT   13
IMRS_RE1075     -------------AGGCTCCTATACGTAAGCGGCCTAAACTTACACCACCTCAGCCTACT   399
RE1075          -----------------------------------------------TCCTCAGCCTACT   13
                                                                ***** ******


IMRS_RE1132     ATAATGCCTATAAGTACAGCTGATCGTGATATGGGTGTTGATGTTATTAATGAGCCTCAA   480
RE1132          ATAATGCCTATAAGTACAGCTGATCGTGATATGGGTGTTGATGTTATTAATGAGCCTCAA   72
IMRS_RE451      ATAATGCCTATAAGTACAGCTGATCGTGATATGGGTGTTGATGTTATTAATGAGCCTCAA   480
RE451           ATAATGCCTATAAGTATAGCTGATCGTGATGTGGGGGTTGATACTGATATTCTTGCTCAG   73
IMRS_RE773      ATAATGCCTATAAGTATAGCTGATCGTGATGTGGGGGTTGATACTGATATTCTTGCTCAG   459
RE773           ATAATGCCTATAAGTATAGCTGATCGTGATGTGGGGGTTGATACTGATATTCTTGCTCAG   73
IMRS_RE1075     ATAATGCCTATAAGTATAGCTGATCGTGATGTGGGGGTTGATACTGATATTCTTGCTCAG   459
RE1075          ATAATGCCTATAAGTATAGCTGATCGTGATGTGGGGGTTGATACTGATATTCTTGCTCAG   73
                **************** ************* **** ******  *  ** *    ****

IMRS_RE1132     GCTCAAGTAGCACAAGCTCAGCAAAATGATCCTCTTGTTCGTGGATTACGTAGGATTGCT   540
RE1132          GCTCAAGTAGCACAAGCTCAGCAAAATGATCCTCTTGTTCGTGGATTACGTAGGATTGCT   132
IMRS_RE451      GCTCAAGTACCACAAGCTCAGCAAAATGATCCTCTTGTTCGTGGATTACGTAGGATTGCT   540
RE451           GCTGCTGCTGGGCAACCACAGCTTACTGT---TGAGCAGCGTGCTGCAGAGAGGATTGCT   130
IMRS_RE773      GCTGCTGCTGGGCAACCACAGCTTACTGT---TGAGCAGCGTGCTGCAGAGAGGATTGCT   516
RE773           GCTGCTGCTGGGCAACCACAGCTTACTGT---TGAGCAGCGTGCTGCAGAGAGGATTGCT   130
IMRS_RE1075     GCTGCTGCTGGGCAACCACAGCTTACTGT---TGAGCAGCGTGCTGCAGAGAGGATTGCT   516
RE1075          GCTGCTGCTGGGCAACCACAGCTTACTGT---TGAGCAGCGTGCTGCAGAGAGGATTGCT   130
                ***   *     *** * ****  * **    *      ****    *   *********

IMRS_RE1132     TGGTTAAAACAGTATGCTGGTATTGACTATATGGTGAAGGATCCTAATAATCCTGGGCAG   600
RE1132          TGGTTAAAACAGTATGCTGGTATTGACTATATGGTGAAGGATCCTAATAATCCTGGGCAG   192
IMRS_RE451      TGGTTAAAACAGTATGCTGGTATTGACTATATGGTGAAGGATCCTAATAATCCTGGGCAG   600
RE451           TGGTTGAAGAATTATGCTGGTATTGACTATATGGTCCCAGATCCTCAGAATCCTCATGCT   190
IMRS_RE773      TGGTTGAAGAATTATGCTGGTATTGACTATATGGTCCCAGATCCTCAGAATCCTCATGCT   576
RE773           TGGTTGAAGAATTATGCTGGTATTGACTATATGGTCCCAGATCCTCAGAATCCTCATGCT   190
IMRS_RE1075     TGGTTGAAGAATTATGCTGGTATTGACTATATGGTCCCAGATCCTCAGAATCCTCATGCT   576
RE1075          TGGTTGAAGAATTATGCTGGTATTGACTATATGGTCCCAGATCCTCAGAATCCTCATGCT   190
                ***** **  * ***********************    ****** * ******
```

```
IMRS_RE1132      ATGATGGTAAATCCTGTGTTGTTAAATATTCCTCAAGGTCCGCCTGCTAATAATCCAAGA   660
RE1132           ATGATGGTAAATCCTGTGTTGTTAAATATTCCTCAAGGTCCGCCTGCTAATAATCCAAGA   252
IMRS_RE451       ATGATGGTAAATCCTGTGTTGTTAAATATTCCTCAAGGTCCGCCTGCTAATAATCCAAGA   660
RE451            AGAGTTATAAATCCTGTGTTGTTAAATATTACTCAGGGGCCACCTAACGTACAGCCTA--   248
IMRS_RE773       AGAGTTATAAATCCTGTGTTGTTAAATATTACTCAAGGGCCACCTAACGTACAGCCTA--   634
RE773            AGAGTTATAAATCCTGTGTTGTTAAATATTACTCAAGGGCCACCTAACGTACAGCCTA--   248
IMRS_RE1075      AGAGTTATAAATCCTGTGTTGTTAAATATTACTCAGGGGCCACCTAACGTACAGCCTA--   634
RE1075           AGAGTTATAAATCCTGTGTTGTTAAATATTACTCAGGGGCCACCTAACGTACAGCCTA--   248
                 *   *  *********************** **** ** ** ***       * ** *

IMRS_RE1132      GCGCCTATGCAACGTTGTGATATACTTAATCATGATCACTGGAGGCATTTAGTAGTTGGT   720
RE1132           GCGCCTATGCAACGTTGTGATATACTTAATCATGATCACTGGAGGCATTTAGTAGTTGGT   312
IMRS_RE451       GCGCCTATGCAACGTTGTGATATACTTAATCATGATCACTGGAGGCATTTAGTAGTTGGT   720
RE451            -GACCTCGGCAAGATCTTAACATACTTGACCATGCTCAGTGGAGACATTTGGTAGTTGGT   307
IMRS_RE773       -GACCTCGGCAAGATCTTAACATACTTGACCATGCTCAGTGGAGACATTTGGTAGTTGGT   693
RE773            -GACCTCGGCAAGATCTTAACATACTTGACCATGCTCAGTGGAGACATTTGGTAGTTGGT   307
IMRS_RE1075      -GACCTCGGCAAGATCTTAACATACTTGACCATGCTCAGTGGAGACATTTGGTAGTTGGT   693
RE1075           -GACCTCGGCAAGATCTTAACATACTTGACCATGCTCAGTGGAGACATTTGGTAGTTGGT   307
                    ***  ****  *  * * ****** * **** *** ***** ***** *********

IMRS_RE1132      ATTGCTGCATTATCAAATGCTAATAAACCTAGCGCTTCTCCTGTCAAAGTATTAAGTGAT   780
RE1132           ATTGCTGCATTATCAAATGCTAATAAACCTAGCGCTTCTCCTGTCAAAGTATTAAGTGAT   372
IMRS_RE451       ATTGCTGCATTATCAAATGCTAATAAACCTAGCGCTTCTCCTGTCAAAGTATTAAGTGAT   780
RE451            GTTACTGCATTGTCACATGCTAATAAACCTAGCGTTACTCCTGTCAAAGTATTAAGTGAC   367
IMRS_RE773       GTTACTGCATTGTCACATGCTAATAAACCTAGTGTTACTCCTGTCAAAGTATTAAGTGAC   753
RE773            GTTACTGCATTGTCACATGCTAATAAACCTAGTGTTACTCCTGTCAAAGTATTAAGTGAC   367
IMRS_RE1075      GTTACTGCATTGTCACATGCTAATAAACCTAGCGTTACTCCTGTCAAAGTATTAAGTGAC   753
RE1075           GTTACTGCATTGTCACATGCTAATAAACCTAGCGTTACTCCTGTCAAAGTATTAAGTGAC   367
                  ** ******* *** **************** * * **********************

IMRS_RE1132      AAAATTACTCAGATATATAGTGATATAAAGCCATTTGCTGATATAGCTGGTATTGATGTT   840
RE1132           AAAATTACTCAGATATATAGTGATATAAAGCCATTTGCTGATATAGCTGGTATTGATGT-   431
IMRS_RE451       AAAATTACTCAGATATATAGTGATATAAAGCCATTTGCTGATATAGCTGGTATTGATGTT   840
RE451            AAAATTACTAAGATATATAGTGATATAAGACAATTCGCTAAGATAGCTAATATCGAAATT   427
IMRS_RE773       AAAATTACTAAGATATATGGTGATATAAGACAATTCGCTAAGATAGCTAATATCGAAGTT   813
RE773            AAAATTACTAAGATATATGGTGATATAAGACAATTCGCTAAGATAGCTAATATCGAAGTT   427
IMRS_RE1075      AAAATTACTAAGATATATAGTGATATAAGACAATTCGCTAAGATAGCTAATATCGAAATT   813
RE1075           AAAATTACTAAGATATATAGTGATATAAGACAATTCGCTAAGATAGCTAATATCGAAATT   427
                 ********* ******** *********  * *** *** * ******  *** **  *

IMRS_RE1132      CCTGATACTGGTTTGCCTAATAGTGCGTCTGTCGAACAGATACAGAGAAAAATGCAAGAA   900
RE1132           CCTGAT------------------------------------------------------   437
IMRS_RE451       CCTGATACTGGTTTGCCTAATAGTGCATCTGTCGGGATCCCTAGA---------------   885
RE451            CCTGATGCTCCTTTGCCTAATAGTGCATCTGTCGGGATCCCTAGA---------------   472
IMRS_RE773       CCTGATGCTCCTTTGCCTAATAGTGCATCTGTTGAACAGATACAGGCTAAAATGGAAGAA   873
RE773            CCTGATGCTCCTTTGCCTAATAGTGCATCTGT----------------------------   459
IMRS_RE1075      CCTGATGCTCCTTTGCCTAATAGTGCATCTGTTGAACAGATACAGGCTAAAATGGAAGAA   873
RE1075           CCTGATGCTCCTTTGCCTAATAGTGCATCTGT----------------------------   459
                 *
```

Figure S1.(A) Multiple sequence alignment of OT *tsa56* genes for Karp and TA763 genotypes (B) Percent identity analysis of aligned OT *tsa56* genes for Karp and TA763 genotypes. CMCOT7 and CMCOT11 were compared with Karp (blue) and TA763 (green) genotypes (C) Multiple sequence alignment of OT nPCR and single-run PCR generated *tsa56* genes (n=12). The alignments were sectioned by Kato, Karp and TA763 genotypes.
